# *SCN5A* Mutation Type and a Genetic Risk Score Associate Variably With Brugada Syndrome Phenotype in *SCN5A* Families

**DOI:** 10.1161/CIRCGEN.120.002911

**Published:** 2020-11-09

**Authors:** Yanushi D. Wijeyeratne, Michael W. Tanck, Yuka Mizusawa, Velislav Batchvarov, Julien Barc, Lia Crotti, J. Martijn Bos, David J. Tester, Alison Muir, Christian Veltmann, Seiko Ohno, Stephen P. Page, Joseph Galvin, Rafik Tadros, Martina Muggenthaler, Hariharan Raju, Isabelle Denjoy, Jean-Jacques Schott, Jean-Baptiste Gourraud, Doris Skoric-Milosavljevic, Eline A. Nannenberg, Richard Redon, Michael Papadakis, Florence Kyndt, Federica Dagradi, Silvia Castelletti, Margherita Torchio, Thomas Meitinger, Peter Lichtner, Taisuke Ishikawa, Arthur A.M. Wilde, Kazuhiro Takahashi, Sanjay Sharma, Dan M. Roden, Martin M. Borggrefe, Pascal P. McKeown, Wataru Shimizu, Minoru Horie, Naomasa Makita, Takeshi Aiba, Michael J. Ackerman, Peter J. Schwartz, Vincent Probst, Connie R. Bezzina, Elijah R. Behr

**Affiliations:** 1 Molecular and Clinical Sciences Research Institute, St George’s University of London, Cardiovascular Clinical Academic Group, St George’s University Hospitals National Health Service (NHS) Foundation Trust, United Kingdom (Y.D.W., V.B., M.M., H.R., M.P., S.S., E.R.B.).; 2 European Reference Network for Rare & Low Prevalence Complex Diseases of the Heart (ERN GUARD-Heart) (Y.D.W., Y.M., V.B., J.B., L.C., R.T., M.M., H.R., J.-J.S., J.-B.G., D.S.-M., E.A.N., R.R., M.P., F.K., F.D., S.C., M.T., A.A.M.W., S.S., P.J.S., V.P., C.R.B., E.R.B.).; 3 Departments of Clinical Epidemiology, Biostatistics and Bioinformatics, Amsterdam Public Health (M.W.T.); 4 Heart Center, Department of Clinical and Experimental Cardiology, Amsterdam Cardiovascular Sciences, Amsterdam UMC (Y.M., R.T., D.S.-M., E.A.N., A.A.M.W., C.R.B.), University of Amsterdam, the Netherlands.; 5 l’institut du thorax, INSERM, CNRS, UNIV Nantes, France (J.B., J.-J.S., J.-B.G., R.R., F.K.).; 6 Center for Cardiac Arrhythmias of Genetic Origin and Laboratory of Cardiovascular Genetics (L.C., F.D., S.C., M.T., P.J.S.), Milan, Italy.; 7 Department of Cardiovascular, Neural and Metabolic Sciences, San Luca Hospital and Department of Medicine and Surgery, University of Milano-Bicocca, Istituto Auxologico Italiano, IRCCS, Milan, Italy (L.C.).; 8 Departments of Cardiovascular Medicine (Division of Heart Rhythm Services), Pediatric and Adolescent Medicine (Division of Pediatric Cardiology), and Molecular Pharmacology and Experimental Therapeutics (Windland Smith Rice Sudden Death Genomics Laboratory), Mayo Clinic, Rochester, MN (J.M.B., D.J.T., M.J.A.).; 9 Belfast Health & Social Care Trust, United Kingdom (A.M., P.P.M.).; 10 Rhythmology and Electrophysiology, Department of Cardiology and Angiology, Hannover Medical School, Germany (C.V.).; 11 Shiga University of Medical Science (S.O., M.H.).; 12 National Cerebral and Cardiovascular Center, Osaka, Japan (S.O., T.I., W.S., N.M., T.A.).; 13 Leeds Teaching Hospitals NHS Trust, United Kingdom (S.P.P.).; 14 Mater University and Private Hospitals, Dublin, Ireland (J.G.).; 15 AP-HP, Hôpital Bichat, Dépt de Cardiologie et Ctr de Référence des Maladies Cardiaques Héréditaires, Univ Paris Diderot, Sorbonne Paris Cité, Paris, France INSERM U1166 (I.D.).; 16 CHU Nantes, Service de Génétique Médicale (J.-J.S., J.-B.G., R.R.).; 17 l’institut du thorax, CHU Nantes, Service de Cardiologie, Nantes, France (F.K.).; 18 Helmholtz Zentrum München, Institute of Human Genetics, Neuherberg (T.M., P.L.).; 19 Technische Universität München, Institute of Human Genetics (T.M.).; 20 DZHK (German Center for Cardiovascular Research), Partner Site Munich Heart Alliance, Germany (T.M.).; 21 Kizawa memorial hospital, Gifu, Japan (K.T.).; 22 Vanderbilt University School of Medicine, Nashville, TN (D.M.R.).; 23 Department of Medicine, University Medical Center Mannheim (UMM), Faculty of Medicine Mannheim, University of Heidelberg, European Center for AngioScience (ECAS) & DZHK (German Center for Cardiovascular Research) partner site Heidelberg/Mannheim, Germany (M.M.B.).; 24 Queen’s University Belfast, United Kingdom (P.P.M.).; 25 Nippon Medical School, Tokyo, Japan (W.S.).; 26 Reference Center for hereditary arrhythmic diseases, Cardiologic Department and INSERM U1087, L’Institut du Thorax, Nantes, France (V.P.).

**Keywords:** Brugada syndrome, genetics, human, penetrance, phenotype, risk score

## Abstract

Brugada syndrome (BrS) is characterized by the type 1 Brugada ECG pattern. Pathogenic rare variants in *SCN5A* (mutations) are identified in 20% of BrS families in whom incomplete penetrance and genotype-negative phenotype-positive individuals are observed. E1784K-*SCN5A* is the most common *SCN5A* mutation identified. We determined the association of a BrS genetic risk score (BrS-GRS) and *SCN5A* mutation type on BrS phenotype in BrS families with *SCN5A* mutations.

**Methods:**

Subjects with a spontaneous type 1 pattern or positive/negative drug challenge from cohorts harboring *SCN5A* mutations were recruited from 16 centers (n=312). Single nucleotide polymorphisms previously associated with BrS at genome-wide significance were studied in both cohorts: rs11708996, rs10428132, and rs9388451. An additive linear genetic model for the BrS-GRS was assumed (6 single nucleotide polymorphism risk alleles).

**Results:**

In the total population (n=312), BrS-GRS ≥4 risk alleles yielded an odds ratio of 4.15 for BrS phenotype ([95% CI, 1.45–11.85]; *P*=0.0078). Among *SCN5A*-positive individuals (n=258), BrS-GRS ≥4 risk alleles yielded an odds ratio of 2.35 ([95% CI, 0.89–6.22]; *P*=0.0846). In *SCN5A*-negative relatives (n=54), BrS-GRS ≥4 alleles yielded an odds ratio of 22.29 ([95% CI, 1.84–269.30]; *P*=0.0146). Among E1784K-*SCN5A* positive family members (n=79), hosting ≥4 risk alleles gave an odds ratio=5.12 ([95% CI, 1.93–13.62]; *P*=0.0011).

**Conclusions:**

Common genetic variation is associated with variable expressivity of BrS phenotype in *SCN5A* families, explaining in part incomplete penetrance and genotype-negative phenotype-positive individuals. *SCN5A* mutation genotype and a BrS-GRS associate with BrS phenotype, but the strength of association varies according to presence of a *SCN5A* mutation and severity of loss of function.

## Abstract 2

Supplemental Digital Content is available in the text.

Brugada syndrome (BrS) is characterized by the type 1 Brugada ECG pattern, present either spontaneously or after provocation with a sodium channel blocking agent.^1^ Pathogenic rare variants (mutations) in the *SCN5A* gene, encoding the Nav1.5 sodium channel, are identified in 20% of cases.^2,3^ Incomplete penetrance and variable expression is common in BrS pedigrees with *SCN5A* mutations, suggesting a complex inheritance wherein other genetic variants may affect the phenotype.^2^ Genotype-negative individuals from *SCN5A*-positive pedigrees have shown the type 1 Brugada ECG pattern.^2^ Furthermore, common genetic variation has been associated with BrS in probands, independent of *SCN5A* status.^4^

The E1784K*-SCN5A* mutation (c.5350G>A; ClinVar ID: 9377) is the most common *SCN5A* mutation identified in BrS, identified in 3% of unrelated BrS cases^3,5^ and is absent in the gnomAD database. Furthermore, E1784K-*SCN5A* exhibits incomplete penetrance and can manifest as a mixed clinical phenotype of long QT syndrome and/or BrS, even among affected individuals from the same pedigree.^6,7^ These properties make E1784K*-SCN5A* an optimal target for studying potential genetic modifiers.^8^

We hypothesized that common genetic variation previously associated with BrS,^4^ and a genetic risk score derived thereof (BrS-genetic risk score [GRS]), is associated with a type 1 Brugada ECG pattern in genotype-positive individuals from BrS families hosting *SCN5A* mutations as well as in genotype-negative relatives. We then explored the effects of *SCN5A* mutation type on the likelihood of a type 1 Brugada ECG pattern.

## Methods

The data that support the findings of this study are available from the corresponding author upon reasonable request. Institutional Review Board approval was obtained, according to the guidelines noted in instructions to authors. The full methods are available as Data Supplement.

## Results

### Clinical Characteristics

**Figure 1.**
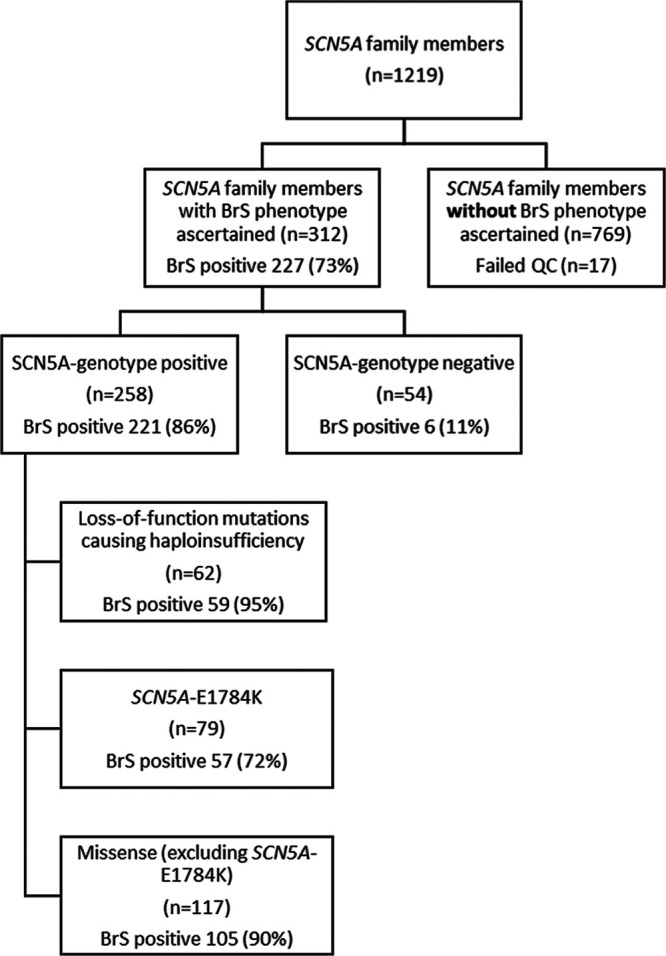
Flow diagram summarizing inclusion and numbers of individuals separated by genotype and Brugada syndrome (BrS) phenotype in each cohort.

The total cohort comprised of 312 individuals from families harboring *SCN5A* mutations. The individuals that fulfilled inclusion criteria had the presence or absence of the BrS phenotype definitively established and had undergone complete single nucleotide polymorphism (SNP) genotyping (Figure 1). These 312 individuals were recruited from 137 families. The median family size was 1 (Q1–Q3: 1–2); 4 families had between 10 and 20 individuals and a single family contributed 31 individuals. Figure 1 illustrates the breakdown of included cases according to *SCN5A* genotype and mutation type.

Clinical characteristics are described and compared in Tables 1 and 2. Subjects hosting *SCN5A*-E1784K, when compared with individuals harboring loss-of-function mutations causing haploinsufficiency and other missense *SCN5A* mutations, were younger and more likely to be female. As would be expected when comparing individuals with an overlap syndrome to those with conduction disease, they exhibited longer QT intervals and shorter PR intervals and QRS durations on their presenting ECGs.

**Table 1.**
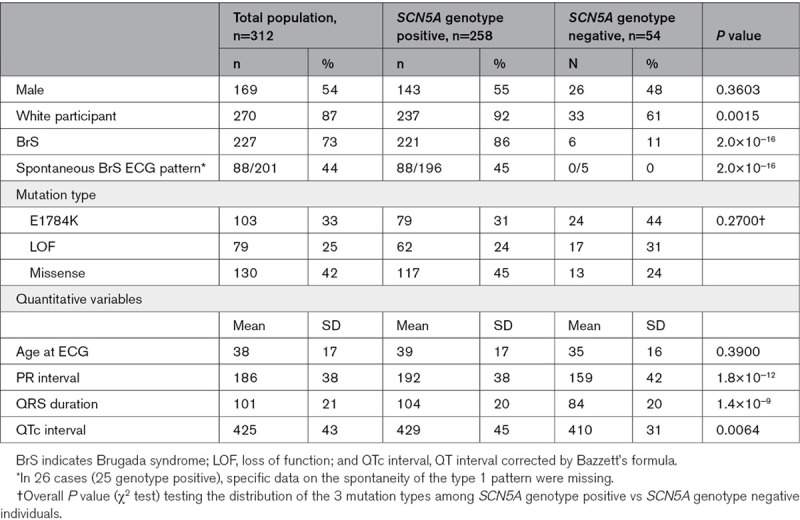
Clinical Characteristics of the Total Cohort Broken Down by Genotype Status

**Table 2.**
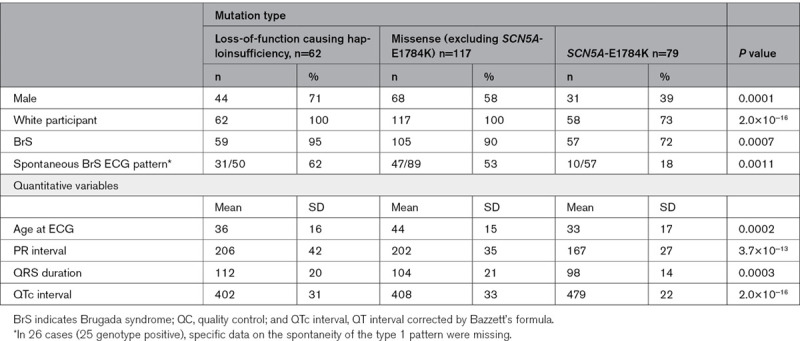
Clinical Characteristics of Individuals Harboring Loss-of-function mutations causing haploinsufficiency, missense mutations excluding *SCN5A*-E1784K, and *SCN5A*-E1784K

Seventy-nine individuals were E1784K*-SCN5A* positive. Fifty-seven (72%) with E1784K*-SCN5A* had BrS phenotype (10 spontaneous; 47 drug-induced). Among the 179 individuals harboring loss-of-function mutations causing haploinsufficiency and other missense *SCN5A* mutations, 164 (92%) had BrS phenotype (78 spontaneous; 61 drug-induced; 25 unspecified). Importantly, 6/54 (17%) *SCN5A*-negative subjects displayed a drug-induced BrS phenotype. The associations of *SCN5A* mutation and/or BrS-GRS with the spontaneous BrS phenotype are similar to those described in both spontaneous and drug induced BrS combined but were less accurate with higher *P* values (data not shown).

### *SCN5A* Mutation Associations

**Figure 2.**
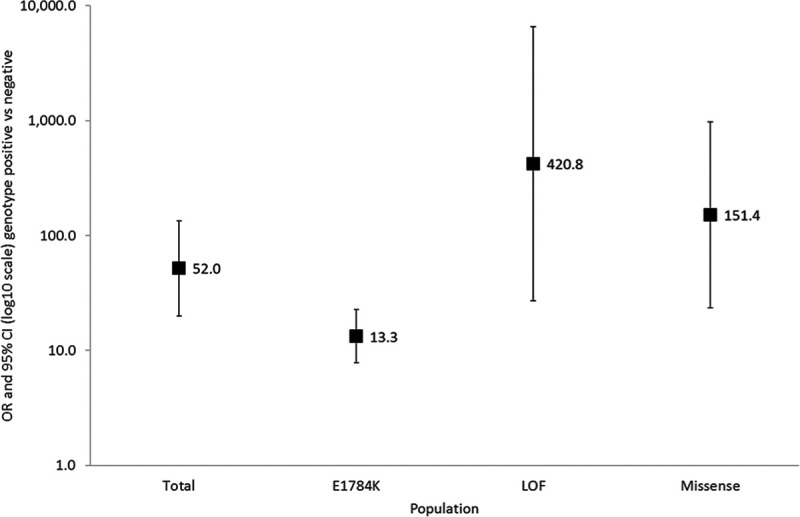
**Risk of Brugada Syndrome in patients carrying an *SCN5A* mutation, loss-of-function (LOF) mutations causing haploinsufficiency, missense mutations other than *SCN5A*, and E1784K-*SCN5A*.** The odds ratio (OR) and 95% CI for each mutation type are shown (adjusted for sex and age). The *P* value denote the levels of significance of the ORs for Brugada Syndrome comparing each cohort to negative genotype using generalized estimating equation.

Among *SCN5A* families, the presence of an *SCN5A* mutation was associated with an odds ratio (OR) of 51.98 ([95% CI, 20.02–134.93], *P*<0.0001) for BrS phenotype (Figure 2). In all 3 *SCN5A* mutation type subgroups, that is, E1784K*-SCN5A*, loss-of-function mutations causing haploinsufficiency and missense mutations other than E1784K*-SCN5A*, genotype positive patients were at an increased risk of BrS compared with genotype negative patients, but the odds ratios differed significantly (*P*_interaction_=0.004) between the mutation types.

Among *SCN5A* genotype positive individuals only, both loss-of-function mutations causing haploinsufficiency and other missense mutations had an increased risk of BrS compared with E1784K*-SCN5A* with OR=6.11 ([95% CI, 1.78–20.97]; *P*=0.0040) and OR=3.44 ([95% CI, 1.35–8.75]; *P*=0.0095), respectively.

### BrS Genetic Risk Score

**Figure 3.**
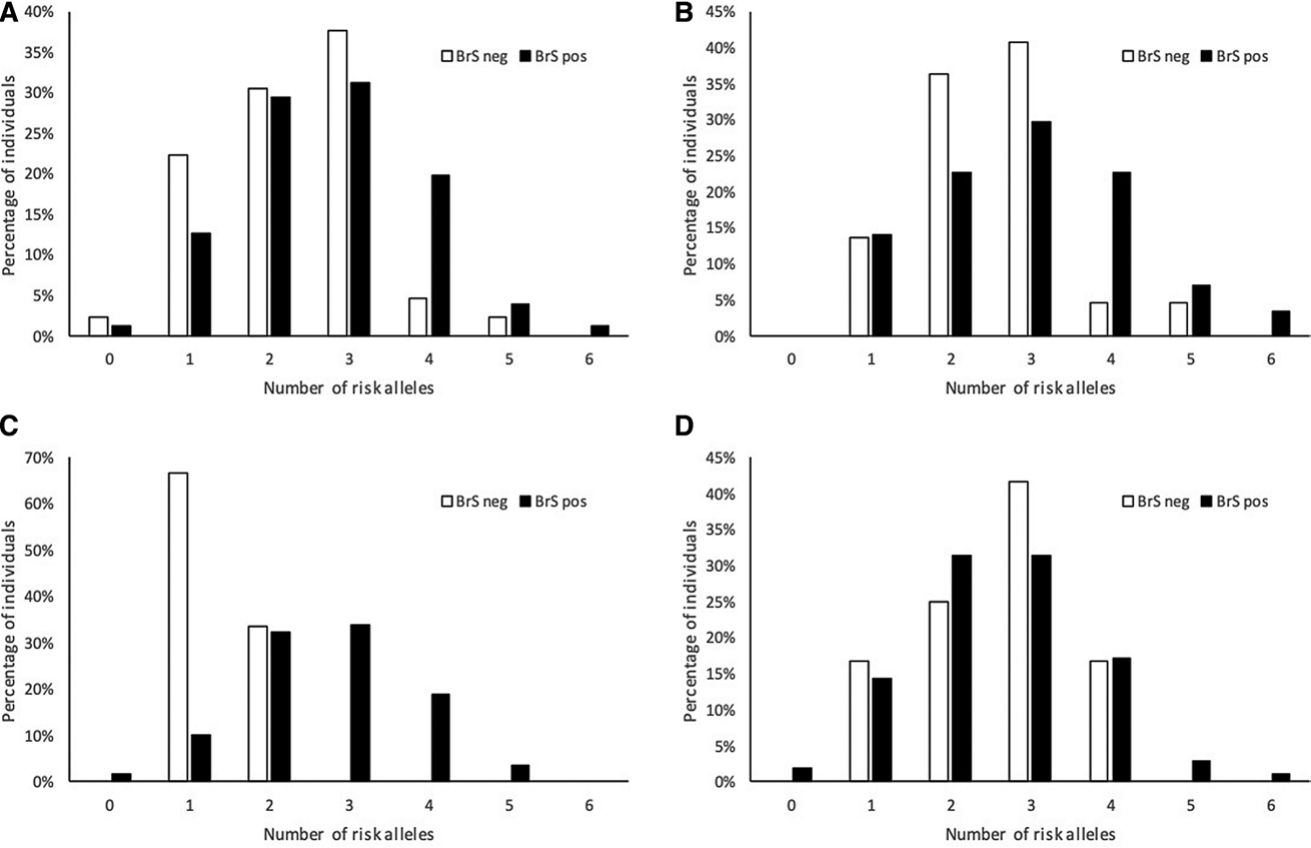
**Cumulative number of risk alleles at the 3 loci and the associated likelihood of Brugada Syndrome (BrS) phenotype showing performance of the BrS-genetic risk score for prediction of BrS phenotype in mutation positive individuals.A**, Total cohort; (**B**) individuals harboring *E1784K*-SCN5A; (**C**) individuals harboring loss-of-function *SCN5A* mutations causing haploinsufficiency; (**D**) individuals harboring other missense *SCN5A* mutations. Distribution of numbers of risk alleles hosted by individuals with BrS phenotype (black bars) in each cohort are shown vs family members ascertained to be BrS phenotype-negative (white bars). Each bar represents the proportion of individuals carrying the corresponding number of risk alleles as a percentage of the total number of individuals with the corresponding phenotype, that is, denominator for the white bars being the total number of individuals with no BrS within the cohort, and the denominator for the black bars being the total number of individuals with BrS within the cohort.

The BrS-GRS was calculated for each subject in the total cohort as described. A weighted BrS-GRS was also tested, but this did not outperform the nonweighted BrS-GRS (data not shown). Figure 3 shows the distribution of proportion of subjects according to numbers of risk alleles (range, 0–6) for the total cohort and subsets of *SCN5A* mutations. In the total population, the odds ratio per allele was 1.46 ([95% CI, 1.11–1.94], *P*=0.0076) and individuals with a BrS-GRS ≥4 risk alleles had an OR=4.15 ([95% CI, 1.45–11.85], *P*=0.0078) for BrS phenotype compared with individuals with a GRS <4 risk alleles.

The BrS-GRS effects per allele and ≥4 risk alleles appeared smaller in *SCN5A* genotype positives, but this was not significant (*P*_interaction_=0.090 and 0.076, respectively). Within *SCN5A* genotype positives only, the BrS-GRS effects per allele and ≥4 risk alleles were significantly different between mutation types (*P*_interaction_=0.0096 and <0.0001, respectively).

**Figure 4.**
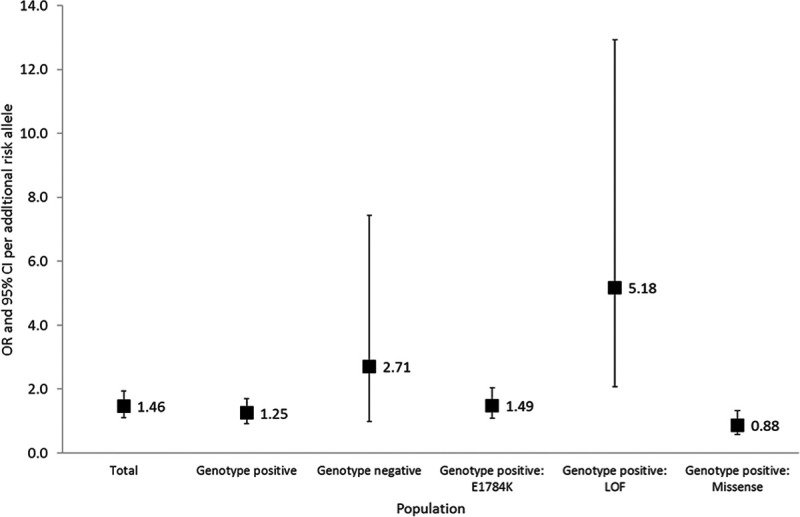
**Risk per additional risk allele in a linear model in the total cohort; genotype negative individuals; genotype positive individuals from families harboring loss-of-function (LOF) mutations causing haploinsufficiency; genotype positive individuals from families harboring E1784K-*SCN5A*; genotype positive individuals from families harboring other missense *SCN5A* mutations.** The odds ratio (OR) per additional risk allele and 95% CI are shown (adjusted for sex and age). The *P* value denotes the levels of significance of the ORs per additional risk allele for Brugada Syndrome in each cohort using generalized estimating equation. The OR and 95% CI for genotype positive: loss-of-function causing haploinsufficiency cohort are not shown as these are off the scale of this figure.

**Figure 5.**
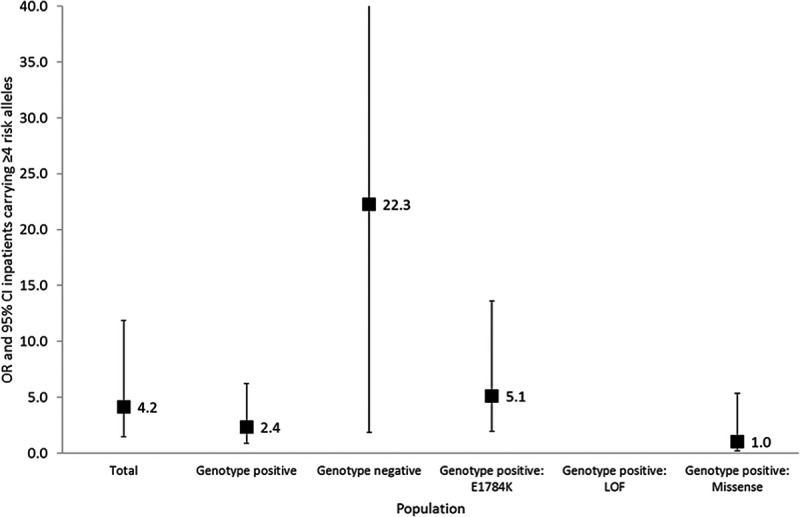
**Risk of Brugada Syndrome in patients carrying ≥4 risk alleles in the total cohort; genotype negative individuals; genotype positive individuals from families harboring loss-of-function mutations causing haploinsufficiency; genotype positive individuals from families harboring E1784K-SCN5A; genotype positive individuals from families harboring other missense SCN5A mutations.** The odds ratio (OR) and 95% CI for a cutoff of ≥4 risk alleles are shown (adjusted for age and sex). The *P* value denotes the level of significance of the ORs for this cutoff for Brugada Syndrome for each cohort using generalized estimating equation.

*SCN5A* genotype-positive relatives (n=258) yielded an OR=1.25 ([95% CI, 0.92–1.71], *P*=0.1571) for BrS phenotype per risk allele. Individuals with a BrS-GRS ≥4 risk alleles had an OR=2.35 ([95% CI, 0.89–6.22], *P*=0.0846) for BrS phenotype compared with individuals with a GRS <4 risk alleles. *SCN5A* genotype-negative relatives (n=54) yielded an OR for BrS phenotype of 2.71 per risk allele ([95% CI, 0.98–7.43]; *P*=0.0535). *SCN5A* genotype-negative individuals with a BrS-GRS ≥4 risk alleles had an OR=22.29 ([95% CI, 1.84–269.30], *P*=0.0146) for BrS phenotype compared with individuals with a BrS-GRS <4 risk alleles (Figures 4 and 5).

### SCN5A Loss-of-Function Mutations Causing Haploinsufficiency

For subjects hosting loss-of-function *SCN5A* mutations causing haploinsufficiency, the association between the BrS-GRS and BrS phenotype appeared the strongest (OR per risk allele of 5.18 [95% CI, 2.07–12.93], *P*=0.0004). As there were no BrS negative cases that had >2 risk alleles, the OR of subjects with ≥4 risk alleles was infinite compared with subjects with <4 risk alleles (Figures 3 through 5).

### SCN5A-E1784K

When examining E1784K*-SCN5A* positive family members alone, a weaker BrS-GRS performance was found: OR=1.49 ([95% CI, 1.09–2.04], *P*=0.0135) per risk allele. Individuals with a BrS-GRS ≥4 risk alleles had an OR=5.12 ([95% CI, 1.93–13.62], *P*=0.0011) for BrS phenotype compared with individuals with a GRS <4 risk alleles (Figures 4 and 5).

### Other Missense SCN5A Mutations

For individuals hosting other *SCN5A* mutations, there was no statistically significant association between the BrS-GRS and BrS phenotype (OR per risk allele=0.88 [95% CI, 0.58–1.32], *P*=0.5271). Subjects with ≥4 risk alleles had an OR=1.03 ([95% CI, 0.20–5.35], *P*=0.9705) for BrS phenotype compared with those with <4 risk alleles (Figures 4 and 5).

## Discussion

Historically, BrS was considered an autosomal dominant monogenic disorder. In 2013, a common variant genome-wide association study (GWAS) comparing index cases of BrS to healthy controls indicated association with common genetic variation, regardless of presence of an *SCN5A* mutation.^4^ While that work identified susceptibility loci, up to now, the variable expression of the BrS phenotype in members of families with *SCN5A* mutations has remained unexplained. Here, for the first time, we report that common genetic variation, in the form of a BrS-GRS, correlates with the BrS phenotype in individuals from families with *SCN5A* loss-of-function mutations causing haploinsufficiency and the recurrent mutation E1784K-*SCN5A*. Furthermore, our study extends beyond the findings of the original GWAS by emphasizing the role of common variation in expression of the BrS phenotype independent of the presence of an *SCN5A* mutation. The BrS-GRS explained in part the variable expression of BrS phenotype in both *SCN5A*-positive and *SCN5A*-negative relatives. There was significant heterogeneity of the strength of association of different types of *SCN5A* mutation (loss-of-function causing haploinsufficiency, E1784K, and other missense) and their associated BrS-GRS with BrS phenotype indicating a variable biological effect of common and rare variants on disease susceptibility. These findings support a complex polygenic architecture for BrS and are an important proof of principle in cardiac genetics.

### A BrS-GRS and Variability in BrS Phenotype Within Affected Families

We sought to investigate whether a BrS-GRS is associated with BrS phenotype. The score demonstrated association with BrS phenotype in pedigrees carrying pathogenic or likely pathogenic *SCN5A* variants, reflecting the cumulative effect of the 3 SNPs (6 risk alleles) on BrS phenotype. The BrS-GRS was then tested separately in the subset of families harboring loss-of-function *SCN5A* mutations causing haploinsufficiency, detecting a strong effect size and a near infinite OR when harboring 4 or more risk alleles. This may reflect the small numbers of Brugada negative cases with loss-of-function mutations causing haploinsufficiency and that chromosome 3 risk alleles in trans with the mutant allele are more likely to have a more potent effect by further altering the expression of already haplo-insufficient wild-type *SCN5A*.

Families with missense *SCN5A* mutations other than E1784K-*SCN5A* showed no significant associations with the BrS-GRS while the E1784K-*SCN5A* subset exhibited a significant association, albeit weaker than for loss-of-function mutations causing haploinsufficiency. The reasons for this difference are likely to be complex. First, E1784K-*SCN5A* is considered a relatively mild missense mutation in its biophysical and clinical consequences and showed lower penetrance in our study compared with other missense mutations (72% versus 90%, respectively, Tables 1 and 2).^9^ The association of the BrS-GRS may therefore reflect a greater impact of common variation in this setting. Second, the diversity of the other included missense *SCN5A* mutations may have led to a weaker power for evaluating the BrS-GRS compared with E1784K-SCN5A families. Each mutation is expected to have different severity of biophysical defects with the potential for variable effects of SNPs on the lesion. Furthermore, because of the small size and heterogeneity of the total cohort, there was insufficient power to analyze chromosomal phasing between *SCN5A* mutations and the SNPs of interest. The other missense *SCN5A* mutation group was therefore a less homogeneous group to test for associations than a large group of families with a single mutation such as E1784K-*SCN5A*. More homogeneous samples, particularly founder populations, may be more appropriate for future studies of how common variants modify phenotype. Interestingly in *SCN5A* genotype-negative relatives, the association of BrS-GRS ≥4 risk alleles with BrS phenotype was even more apparent. In fact, the OR was greater than that of E1784K-*SCN5A* in isolation. This supports a greater strength of association of common variation with the likelihood of BrS phenotype in the absence of a *SCN5A* mutation.

These results therefore reveal the potential for clinical utility of incorporating common genetic variation in the form of a genetic risk score in genetic diagnostics for rare disease. It is expected, however, that additional SNPs underlie the complex genetic nature of BrS and a larger GWAS is needed to identify other common variants that could be incorporated to improve the power of an optimized BrS-GRS for diagnostic purposes. This will also require further investigation of greater numbers of relatives with integration of haplotype structure and detailed knowledge of *SCN5A* variants’ biophysical properties.

### Association of Rare *SCN5A* Variation With BrS Phenotype and Common Variants

While common variation in the form of a BrS-GRS has clear independent association with BrS phenotype, the strongest contribution comes from the presence of an *SCN5A* mutation. However, not all *SCN5A* BrS susceptibility mutations have comparable functional effects. The OR for the BrS phenotype associated with E1784K-*SCN5A* is significantly lower than for other missense *SCN5A* mutations but is the greatest in loss-of-function mutations causing haploinsufficiency. Furthermore, the OR of the BrS-GRS for BrS phenotype varied according to *SCN5A* mutation and was the strongest in genotype negative relatives. This suggests that there may be an interaction and synergy of common and rare variation affecting sodium channel function whereby a certain level of impairment is necessary to achieve a threshold where BrS phenotype can manifest. This further supports a polygenic genetic architecture underlying the condition.^10^

### Genotype-Phenotype Mismatch in BrS and Its Implications

The proposed polygenic model of heritability in BrS may explain the paradox of clinically affected mutation-negative individuals in *SCN5A* families, first demonstrated by Probst et al.^2^ Indeed, 12% of *SCN5A*-negative relatives showed a drug-induced BrS phenotype. Importantly, cascade genetic screening in *SCN5A* pedigrees can result in *SCN5A* genotype-negative relatives being discharged from further follow-up. A small proportion of these individuals may still be at risk of developing a BrS phenotype. Conversely, these findings also raise further questions about the specificity of drug provocation tests for BrS in the absence of a gold standard test for the condition. The prevalence of the type 1 Brugada ECG pattern after drug provocation testing has already been shown to be much higher than expected (4%) in healthy controls.^11^ Indeed, recent data have associated a similar BrS polygenic risk score with the ajmaline induced type 1 pattern.^12^ The Shanghai consensus document downgraded the diagnostic certainty offered by such a result when found in isolation.^13^ The likelihood of a drug-induced type 1 Brugada ECG pattern indicating a diagnosis of BrS is considered greater, however, if an individual had a family history of premature autopsy negative sudden cardiac death and/or BrS. The significance of a drug-induced type 1 Brugada ECG pattern in *SCN5A* genotype-negative relatives is therefore uncertain in *SCN5A* BrS families. Other, as yet unknown, polygenic and acquired contributions to the risk of developing BrS phenotype may be present in these *SCN5A* genotype-negative relatives.

BrS phenotype-positive *SCN5A* genotype-negative individuals may be identified because of clinical evaluation taking place either before genetic studies being available, or before determination of the pathogenicity of a detected rare *SCN5A* variant. There is insufficient follow-up data available, however, in the literature to determine if these individuals subsequently develop arrhythmic events. In the meantime, these patients may be offered monitoring for evidence of evolving risk and lifestyle advice such as avoidance of prescription sodium channel blocking drugs, cocaine and alcohol intoxication, and treatment of fever.^14^ Asymptomatic *SCN5A*-negative relatives of autopsy-negative sudden cardiac death victims, who go on to have a positive ajmaline test, have been managed with this strategy. During follow-up, a spontaneous type 1 Brugada ECG pattern and/or clinically significant arrhythmic events developed in 17% of these individuals.^15^ This may be a worthwhile approach in BrS *SCN5A* family members, regardless of genotype status, although further prospective research will be required.

### Future Perspective: an Optimized BrS-GRS

There is already strong association of a BrS-GRS ≥4 risk alleles utilizing only 3 SNPs with BrS phenotype. We propose that an optimized BrS-GRS employing additional SNPs emerging from a larger GWAS could act as a complementary approach to quantifying the probability of developing BrS phenotype. Furthermore, incorporating phasing of SNPs could further refine the predictive accuracy of a BrS-GRS, especially in *SCN5A* families where SNPs in trans to the *SCN5A* mutant allele would be expected to have more pronounced effects than SNPs in cis. An optimized and validated GRS may therefore aid decision-making over follow-up in *SCN5A* families and determining whether preventative and monitoring strategies for BrS should be instituted.^1,13^ A GRS-based approach may even replace the unnecessary use of drug challenge and form part of clinical genetic testing in BrS.

### Limitations

BrS phenotype was defined in accordance with the 2013 Heart Rhythm Society/European Heart Rhythm Association/Asia Pacific Heart Rhythm Society guidelines. Other guidelines have been proposed due to concerns over the specificity of the sodium channel blocker-induced BrS phenotype.^1,13,15^ These guidelines maintain the same definition of the type 1 Brugada ECG pattern and give extra weight to a family history of BrS. We therefore used the same ECG definition for BrS phenotype in this study. We also treated spontaneous and drug-induced BrS phenotype as one group for analysis purposes. This was due to low numbers, the similarity of findings in spontaneous BrS (data not shown) as well as the consistency of the association demonstrated by the BrS GWAS regardless of whether the phenotype was drug-induced or spontaneous.^4^

A smaller proportion of the *SCN5A* genotype-negative cases underwent sodium channel blocker challenge, probably reflecting variation in local clinical practice. Furthermore, only a relatively small proportion of *SCN5A* genotype-positive relatives were found to be BrS phenotype-negative after drug challenge. Both factors likely weakened the power to detect associations.

Because of the heterogeneity of the total cohort, there was insufficient power to analyze chromosomal phasing between the *SCN5A* mutations and the SNPs of interest at this chromosomal locus—rs11708996 (*SCN5A*) and rs10428132 (*SCN10A*)—and therefore SNP interactions. These potential interactions may explain why the weighted model for the BrS-GRS did not show additional significance over the additive model. Furthermore, families of Japanese and other non-White ancestry were included but due to small numbers could not be analyzed separately. This was offset, however, by the 3 SNPs used to create the BrS-GRS having been replicated in Japanese BrS cases.^16^

### Conclusions

Common genetic variation explains in part, the variable expression of BrS phenotype in families with sodium channel disease. Association of common variants was cumulative leading to a BrS-GRS associated with BrS phenotype in both genotype positive and negative subjects, that is, independent of the presence of an *SCN5A* mutation. *SCN5A* mutations and the BrS-GRS also show differing effect sizes on BrS phenotype according to variant type, further confirming a complex polygenic architecture underlying BrS. These findings have important implications in BrS *SCN5A* families where a *SCN5A*-negative relative may still develop a BrS phenotype. Further work is required to elucidate other genetic factors to develop an optimized BrS-GRS that may become a surrogate marker for BrS phenotype in *SCN5A* families, form part of clinical genetic testing, obviate drug provocation testing, and guide follow-up.

## Acknowledgments

The authors would like to acknowledge coauthor Velislav Batchvarov, MD, PhD, who passed away on July 18, 2019.

## Sources of Funding

This work was supported by James Lancaster Memorial Fund sponsored by McColl’s RG Ltd. Y.D. Wijeyeratne and Dr Behr acknowledge funding and ongoing support from the James Lancaster Memorial Fund sponsored by McColl’s RG Ltd. Y.D. Wijeyeratne had received support through an Academic Clinical Fellowship from the National Institute of Health Research. Dr Behr is supported by the Higher Education Funding Council for England and the British Heart Foundation (BHF). Drs Behr, Muggenthaler, Raju, Papadakis, and S. Sharma acknowledge support from Cardiac Risk in the Young. Drs Behr and Raju acknowledge support from BHF Project Grant PG/15/107/31908 and BHF Clinical Research Training Fellowship FS/11/71/28918, respectively. Dr Muggenthaler acknowledges support from the Medical Research Council. Drs Bezzina and Wilde acknowledge the support from the Dutch Heart Foundation (CVON PREDICT2 project to Drs Bezzina and Wilde, HLT) and the Netherlands Organization for Scientific Research (VICI fellowship, 016.150.610, to Dr Bezzina). Dr Tadros received support from the Philippa and Marvin Carsley Cardiology Chair and is currently a clinical research scholar of the Fonds de Recherche du Québec—Santé. Drs Tester, Bos, and Ackerman are supported by the Mayo Clinic Windland Smith Rice Comprehensive Sudden Cardiac Death Program.

## Disclosures

Dr Behr received prior research funds from Biotronik and consulting for Medtronic. Dr Ackerman reports involvement in AliveCor, Audentes Therapeutics, Blue Ox Health, Boston Scientific, Gilead Sciences, Invitae, Medtronic, MyoKardia, StemoniX, and St. Jude Medical. The other authors report no conflicts.

## Supplementary Material


