# Supplementary material for: SCN5A Mutation Type and a Genetic Risk Score Associate Variably With Brugada Syndrome Phenotype in SCN5A Families
Source: Circ Genom Precis Med. 2020 Nov 9;13(6):e002911. doi: 10.1161/CIRCGEN.120.002911 (PMC7748043; doi:10.1161/CIRCGEN.120.002911)
Supplement: Supplementary file 1 [file hcg-13-e002911-s001.pdf]

SUPPLEMENTAL MATERIAL

Methods

Recruitment and case selection

Sixteen Inherited Cardiac Conditions (ICC) specialist centers from France, Germany, Ireland, Italy, Japan, United Kingdom and USA participated. Probands positive for pathogenic or likely pathogenic *SCN5A* variants were recruited. First- and second-degree relatives, whether hosting the familial *SCN5A* variant or not, were then recruited. We only included subjects (n=312) in whom the presence or absence of the type 1 Brugada ECG pattern had been ascertained as: spontaneous; provoked following drug challenge; or confirmed negative following drug challenge (**Figure 1**).[1]

Clinical evaluation

Presenting 12 lead ECGs and details of drug provocation were collected. BrS phenotype was defined as the presence of either a spontaneous or drug-induced type 1 Brugada ECG pattern in at least one right precordial lead (in the standard or higher intercostal spaces).[1] Intravenous drug provocation tests (either ajmaline 1mg/kg, flecainide 2mg/kg or pilsicainide 1mg/kg) were performed at a pre-determined rate as per local protocol.

Classification of variant pathogenicity and subsets

All variants were curated using CardioClassifier (including paralogue annotation).[2] Variants were annotated further by including published functional data to determine variant class using ACMG criteria.[3] Only pathogenic and likely pathogenic variants were included as mutations for this study (see Supplemental Table 1). Frameshift or splice site variants were defined as ‘loss-of-function’ causing haploinsufficiency. Missense variants were divided into *SCN5A*-E1784K mutations and ‘other missense’ mutations.

SNP selection

Three single nucleotide polymorphisms (SNPs) have been associated previously with BrS phenotype in Caucasian and Japanese cohorts at a genome-wide level of significance: rs11708996 (*SCN5A*), rs10428132 (*SCN10A*), and rs9388451 (*HEY2*) (see **Supplemental Table 2** for general population frequencies).[4] The genes tagged by rs10428132 and rs9388451 have undergone functional evaluation previously.[5–8] rs11708996 and rs9388451 were genotyped in all individuals. In families harboring *SCN5A* mutations other than *SCN5A*-E1784K, rs6800541 formed part of a multi-SNP assay previously carried out in this cohort and was found to be in complete linkage disequilibrium with rs10428132 ($r^2 = 1$). It was therefore used as a proxy SNP.

SNP genotyping

SNP genotyping in *SCN5A*-E1784K families was carried out using the PCR-based Kompetitive Allele Specific PCR (KASP™) genotyping assay provided by LGC, UK.[9] The KASP™ assays were designed using the Kraken™ software system and validated through LGC's assay validation pipeline.[10] Quality control post-genotyping included exclusion of samples where complete data were not available for all three SNPs.

Genotyping of the three SNPs in the rest of the cases was performed as part of a multi-SNP assay on the MassARRAY system using matrix-assisted laser desorption ionization time-of-flight mass spectrometry with iPLEX™ Gold chemistry (Sequenom Inc, San Diego, CA, USA). Primers were designed using Assay Designer 4.0.0.2 with iPLEX Gold default parameters. Automated genotype calling was done with MassArrayTyper (version 4.0.22.67). An experienced evaluator checked genotype clustering visually. We excluded samples where the overall SNP call rate was < 95% or where the genotype for any of the three SNPs was missing.

Statistical analyses

Categorical variables are described as count and percentage and numerical variables as mean ± standard deviation (SD) or median and interquartile range (Q1-Q3: IQR). Categorical variables were compared using Chi-squared and Fisher exact tests where appropriate and numerical variables were compared using ANOVA. For SNP effects, an additive genetic model was assumed. The BrS-GRS was calculated

by summing the number of risk alleles for the three included SNPs that each person carried (range 0-6 alleles). The weighted BrS-GRS was calculated by multiplying the individual risk allele counts with their respective associated effects size (β). The risk alleles and associated effect sizes were assigned based on literature.[5] The weighted BrS-GRS was also tested, but this did not outperform the non-weighted BrS-GRS (data not shown). The assumption of a linear relationship between the BrS-GRS and the natural logarithm of the odds ratio was tested by comparing the fit of the linear model with that of a more flexible model with restricted cubic splines. No significant deviation from linearity was found. Next to a model with the BrS-GRS as a numerical predictor, we also tested the association with a dichotomized predictor (BrS-GRS ≥ 4 yes/no) based on the distribution of risk alleles seen in the overall cohort. All comparisons between BrS phenotype-negative and -positive individuals were carried out using generalized linear models and corrected for sex and age. Generalized estimation equations (GEE), with an independence correlation structure and robust sandwich variance estimations were used to correct for relatedness (geepack, version 1.2-1).[11] The BrS-GRS thus developed was then examined in the total population and subsets of family members harboring *SCN5A* mutations: loss-of-function causing haploinsufficiency; *SCN5A*-E1784K; and other missense mutations. *SCN5A* negative relatives were then studied separately. Interaction terms were added to the models to check for differences in mutation/GRS effects in genotype negative vs. positive and among genotype positive subgroups. Haplotype analyses were not feasible due to small sample sizes.

All analyses were carried out using R (version 3.4.2)[12] and $p<0.05$ was considered statistically significant. Given that this was a candidate SNP study involving three loci, a Bonferroni correction was not considered appropriate.

Ethical approval/Consent

The study was approved by the West London Research Ethics Committee and the St George’s, University of London and St George’s Hospital NHS Trust Joint Research and Enterprise Office. Local research ethics committee approval was obtained for participants recruited from other sites. Informed consent was obtained from all study participants.

Supplemental Tables

Variant (cDNA)	Variant (protein)	gnomAD allele frequency	No BrS phenotype	BrS phenotype	Total
c.1036G>T	p.E346*	0	0	1	1
c.1100G>A	p.R367H	0	0	3	3
c.1100G>T	p.R367L	0	0	1	1
c.1106T>A	p.M369K	0	1	2	3
c.1127G>A	p.R376H	3.2E-05	0	2	2
c.1540G>T	p.G514C	0	0	2	2
c.1603C>T	p.R535*	4.1E-06	11	6	17
c.1657G>T	p.E553*	0	0	1	1
c.1672delC	p.H558Tfs*65	0	0	1	1
c.1890G>A	Splice	5.8E-06	1	2	3
c.1936delC	p.Q646Rfs*5	0	0	1	1
c.1983_1993dup	p.A665Gfs*16	0	5	1	6
c.217C>T	p.Q73*	0	0	1	1
c.2254G>A	p.G752R	4.1E-06	4	6	10
c.2320delT	p.Y774Tfs*28	0	0	2	2
c.2335C>T	p.Q779*	0	0	1	1
c.2373_2374insA	p.S792Kfs*160	0	0	1	1
c.2423G>C	p.R808P	0	0	2	2
c.2516T>C	p.L839P	0	3	5	8
c.2559delT	p.F853Lfs*16	0	0	1	1
c.2582_2583delTT	p.F861Wfs*90	0	0	12	12
c.2632C>T	p.R878C	0	0	2	2
c.2674T>A	p.F892I	0	0	2	2
c.2678G>A	p.R893H	4.1E-06	0	2	2
c.2729C>T	p.S910L	4.1E-06	0	1	1
c.2863G>T	p.E955*	0	0	1	1
c.2894G>A	p.R965H	8.2E-06	0	1	1
c.2943C>A	p.C981*	0	0	2	2
c.3005_3012delCCAGCTGC	p.P1002Hfs*25	0	0	1	1
c.3142_3143insTG	p.P1048Lfs*98	0	0	2	2
c.3175C>T	p.Q1059*	0	1	2	3
c.3352C>T	p.Q1118*	0	0	3	3
c.3491dup	p.E1165Rfs*6	0	0	1	1
c.361C>T	p.R121W	0	0	1	1
c.3622G>T	p.E1208*	0	0	1	1
c.3667delG	p.A1223Pfs*7	0	1	3	4
c.3673G>A	p.E1225K	4.1E-06	0	2	2
c.3676G>T	p.D1226Y	0	0	1	1
c.3694C>T	p.R1232W	0	2	7	9
c.3823G>A	p.D1275N	8.1E-06	0	1	1

c.3956G>T	p.G1319V	4.2E-05	0	5	5
c.3988G>A	p.A1330T	0	0	1	1
c.3G>C	p.M1I	0	0	1	1
c.4030T>C	p.F1344L	0	0	1	1
c.4079T>G	p.F1360C	0	0	1	1
c.4140C>G	p.N1380K	0	0	1	1
c.4213G>A	p.V1405M	0	0	3	3
c.4213G>C	p.V1405L	0	2	3	5
c.4216G>C	p.G1406R	0	2	2	4
c.4222G>A	p.G1408R	0	3	7	10
c.4226A>G	p.Y1409C	0	0	1	1
c.4346A>G	p.Y1449C	0	0	1	1
c.4385T>A	p.L1462Q	0	0	1	1
c.4387A>T	p.N1463Y	0	1	0	1
c.4642G>A	p.E1548K	3.2E-05	0	2	2
c.4719C>T	p.G1573G	3.2E-05	1	1	2
c.4747C>T	p.R1583C	8.1E-06	0	1	1
c.481G>A	p.E161K	8.4E-06	1	9	10
c.4886G>A	p.R1629Q	1.2E-05	0	1	1
c.4894C>T	p.R1632C	4.1E-06	0	2	2
c.4912C>T	p.R1638*	1.2E-05	0	2	2
c.4930C>T	p.R1644C	4.1E-06	0	2	2
c.4952_4953insT	p.M1651Ifs*138	0	0	1	1
c.4958C>T	p.S1653F	0	0	1	1
c.4978A>G	p.I1660V	3.2E-05	1	1	2
c.4981G>A	p.G1661R	0	0	1	1
c.5083C>T	p.Q1695*	0	0	1	1
c.5102T>C	p.M1701T	0	0	1	1
c.511G>A	p.E171K	0	0	1	1
c.5126C>T	p.T1709M	4.1E-06	0	1	1
c.5155C>A	p.P1719T	0	0	2	2
c.5182T>C	p.C1728R	0	1	1	2
c.5228G>A	p.G1743E	0	2	8	10
c.5265C>A	p.Y1755*	0	0	1	1
c.5324delT	p.F1775Sfs*12	0	0	2	2
c.5356_5357delCT	p.L1786Efs*2	0	0	1	1
c.535C>T	p.R179*	4.1E-06	0	1	1
c.5417_5420delCTCA	p.T1806Sfs*27	0	0	1	1
c.5433T>G	p.Y1811*	0	0	1	1
c.617delC	p.T206Kfs*22	0	0	1	1
c.812_813insAGCT	p.F272Afs*40	0	0	1	1

Supplemental Table 1. List of *SCN5A* mutations in the secondary cohort with GnomAD allele frequencies (AF) and breakdown by BrS phenotype.

SNP	Chromosome	Position	Gene/ nearest gene	Alleles	Risk allele	Reported odds ratio	Frequency of the risk allele in the general population (1000 Genomes)[1]
rs11708996[2]	3	intron	SCN5A	G/C	C	1.73	0.0901
rs10428132[2]	3	intron	SCN10A	G/T	T	2.66	0.2815
rs9388451[2,3]	6	intergenic	HEY2-NCOA7	C/T	C	1.58	0.4133
rs6800541	3	intron	SCN10A	C/T	C	NA	0.2420

Supplemental Table 2. List of SNPs evaluated.[4–6] The proxy SNP rs6800541 (highlighted) was in complete linkage disequilibrium (r^2=1.000) with rs10428132. NA= not applicable.

Supplemental References

1. Priori SG, Wilde AA, Horie M, Cho Y, Behr ER, Berul C, Blom N, Brugada J, Chiang C-E, Huikuri H, et al. HRS/EHRA/APHRS expert consensus statement on the diagnosis and management of patients with inherited primary arrhythmia syndromes: document endorsed by HRS, EHRA, and APHRS in May 2013 and by ACCF, AHA, PACES, and AEPC in June 2013. *Heart Rhythm*. 2013;10:1932–1963.
2. Whiffin N, Walsh R, Govind R, Edwards M, Ahmad M, Zhang X, Tayal U, Buchan R, Midwinter W, Wilk AE, et al. CardioClassifier: disease- and gene-

specific computational decision support for clinical genome interpretation. *Genet Med*. 2018;20:1246–1254.

3. Denham NC, Pearman CM, Ding WY, Waktare J, Gupta D, Snowdon R, Hall M, Cooper R, Modi S, Todd D, et al. Systematic re-evaluation of SCN5A variants associated with Brugada syndrome. *J Cardiovasc Electrophysiol*. 2019;30:118–127.
4. Bethesda (MD): National Center for Biotechnology Information NL of M. Database of Single Nucleotide Polymorphisms (dbSNP). dbSNP Build ID 151. [cited 2020 Sep 17]. Available from: https://www.ncbi.nlm.nih.gov/snp
5. Bezzina CR, Barc J, Mizusawa Y, Remme CA, Gourraud J-B, Simonet F, Verkerk AO, Schwartz PJ, Crotti L, Dagradi F, et al. Common variants at SCN5A-SCN10A and HEY2 are associated with Brugada syndrome, a rare disease with high risk of sudden cardiac death. *Nat Genet*. 2013;45:1044–1049.
6. Veerman CC, Podliesna S, Tadros R, Lodder EM, Mengarelli I, de Jonge B, Beekman L, Barc J, Wilders R, Wilde AAM, et al. The Brugada Syndrome Susceptibility Gene HEY2 Modulates Cardiac Transmural Ion Channel Patterning and Electrical Heterogeneity. *Circ Res*. 2017;121:537–548.
7. van den Boogaard M, Smemo S, Burnicka-Turek O, Arnolds DE, van de Werken HJG, Klous P, McKean D, Muehlschlegel JD, Moosmann J, Toka O, et al. A common genetic variant within SCN10A modulates cardiac SCN5A expression. *J Clin Invest*. 2014;124:1844–1852.
8. van den Boogaard M, Wong LYE, Tessadori F, Bakker ML, Dreizehnter LK, Wakker V, Bezzina CR, ’t Hoen PAC, Bakkers J, Barnett P, et al. Genetic variation in T-box binding element functionally affects SCN5A/SCN10A

enhancer. *J Clin Invest*. 2012;122:2519–2530.

9. LGC. Genotyping manuals: KASP Genotyping. 2018 [cited 2020 Sep 17]. Available from: https://www.lgcgroup.com/our-science/genomics-solutions/resource-centre/genotyping/manuals-user-guides/#.WumI-U2ouUl.
10. LGC. Kraken Genotyping Software [cited 2020 Sep 17]. Available from: https://www.lgcgroup.com/products/genotyping-software/kraken/#.Ww6-lk2osy8.
11. Halekoh U, Hojsgaard S, Jun Y. The R Package geepack for Generalized Estimating Equations. *J Stat Softw*. 2006;15:1–11.
12. R Core Team. R: A language and environment for statistical computing. 2017 [cited 2020 Sep 17] Available from: https://www.r-project.org/.
