# Supplementary material for: SCN5A Mutation Type and a Genetic Risk Score Associate Variably With Brugada Syndrome Phenotype in SCN5A Families
Source: Circ Genom Precis Med. 2020 Nov 9;13(6):e002911. doi: 10.1161/CIRCGEN.120.002911 (PMC7748043; doi:10.1161/CIRCGEN.120.002911)
Supplement: Supplementary file 2 [file hcg-13-e002911-s002.pdf]

DOI: 10.1161/CIRCGEN.120.002911

SCN5A Mutation Type and a Genetic Risk Score Associate Variably with Brugada Syndrome Phenotype in *SCN5A* Families

Running title: *Wijeyeratne & Tanck et. al; A Genetic Risk Score associates with BrS phenotype*

Yanushi D. Wijeyeratne, BMedSci, BMBS[1,2]*; Michael W. Tanck, PhD[3]*; Yuka Mizusawa, MD, PhD[2,4]; Velislav Batchvarov, MD, PhD[1,2†]; Julien Barc, PhD[2,5]; Lia Crotti, MD, PhD[2,6,7]; J Martijn Bos, MD, PhD[8]; David J. Tester, PhD[8]; Alison Muir, MD[9]; Christian Veltmann, MD[10]; Seiko Ohno, MD, PhD[11,12]; Stephen P. Page, MD[13]; Joseph Galvin, MD[14]; Rafik Tadros, MD, PhD[2,4]; Martina Muggenthaler, MBBS[1,2]; Hariharan Raju, MBBS, PhD[1,2]; Isabelle Denjoy, MD[15]; Jean-Jacques Schott, PhD[2,5,16]; Jean-Baptiste Gourraud, MD, PhD[2,5,16]; Doris Skoric-Milosavljevic, PhD[2,4]; Eline A. Nannenberg, MD, PhD[2,4]; Richard Redon, PhD[2,5,16]; Michael Papadakis, MBBS, MD[1,2]; Florence Kyndt, PhD[2,5,17]; Federica Dagradi, PhD[2,6]; Silvia Castelletti, MD[2,6]; Margherita Torchio, BSc[2,6]; Thomas Meitinger, MD[18-20]; Peter Lichtner, PhD[18]; Taisuke Ishikawa, PhD[12]; Arthur A.M. Wilde MD, PhD[2,4]; Kazuhiro Takahashi, MD[21]; Sanjay Sharma, MBChB, MD[1,2]; Dan M. Roden, MD[22]; Martin M. Borggrefe, MD, PhD[23]; Pascal P. McKeown, MD[9,24]; Wataru Shimizu, MD, PhD[12,25]; Minoru Horie, MD, PhD[11]; Naomasa Makita, MD, PhD[12]; Takeshi Aiba, MD, PhD[12], Michael J. Ackerman, MD, PhD[8]; Peter J. Schwartz, MD, PhD[2,6]; Vincent Probst, MD, PhD[2,26]; Connie R. Bezzina PhD[2,4]*; Elijah R. Behr MBBS, MD[1,2]*

[1]Molecular & Clinical Sciences Rsrch Inst, St George's Univ of London, Cardiology Clinical Academic Group, St George's Univ Hospitals Nat Health Service (NHS) Foundation Trust, London, UK; [2]European Reference Network for Rare & Low Prevalence Complex Diseases of the Heart (ERN GUARD-Heart); [3]Depts of Clinical Epidemiology, Biostatistics & Bioinformatics, Amsterdam Public Health, [4] Heart Center, Dept of Clinical & Experimental Cardiology, Amsterdam Cardiovascular Sciences, Amsterdam UMC, Univ of Amsterdam, Amsterdam, the Netherlands; [5]l'institut du thorax, INSERM, CNRS, UNIV Nantes, Nantes, France; [6]Ctr for Cardiac Arrhythmias of Genetic Origin & Lab of Cardiovascular Genetics, [7]Dept of Cardiovascular, Neural & Metabolic Sciences, San Luca Hospital & Dept of Medicine & Surgery, Univ of Milano-Bicocca, Istituto Auxologico Italiano, IRCCS, Milan, Italy; [8]Depts of Cardiovascular Medicine (Div of Heart Rhythm Services), Pediatric & Adolescent Medicine (Div of Pediatric Cardiology), & Molecular Pharmacology & Experimental Therapeutics (Windland Smith Rice Sudden Death Genomics Lab), Mayo Clinic, Rochester MN; [9]Belfast Health & Social Care Trust, Belfast, UK; [10]Rhythmology & Electrophysiology, Dept of Cardiology & Angiology, Hannover Medical School, Hannover, Germany; [11]Shiga Univ of Medical Science, Shiga; [12]Nat Cerebral & Cardiovascular Ctr, Osaka, Japan; [13]Leeds Teaching Hospitals NHS Trust, Leeds, UK; [14]Mater Univ & Private Hospitals, Dublin, Ireland; [15]AP-HP, Hôpital Bichat, Dépt de Cardiologie et Ctr de Référence des Maladies Cardiaques Héréditaires, Univ Paris Diderot, Sorbonne Paris Cité, Paris, France INSERM U1166; [16]CHU Nantes, Service de Génétique Médicale; [17]l'institut du thorax, CHU Nantes, Service de Cardiologie, Nantes, France; [18]Helmholtz Zentrum München, Inst of Human Genetics, Neuherberg; [19]Technische Universität München, Inst of Human Genetics; [20]DZHK (German Ctr for Cardiovascular Rsrch), Partner Site Munich Heart Alliance, Munich, Germany; [21]Kizawa memorial hospital, Gifu, Japan; [22]Vanderbilt Univ School of Medicine, Nashville, TN; [23]Dept of Medicine, Univ Medical Ctr Mannheim (UMM), Faculty of Medicine Mannheim, Univ of Heidelberg, European Ctr for AngioScience (ECAS) & DZHK (German Ctr for Cardiovascular Rsrch) partner site Heidelberg/Mannheim, Mannheim, Germany; [24]Queen's Univ Belfast, Belfast, UK; [25]Nippon Medical School, Tokyo, Japan; [26]L'Institut du Thorax, Reference Ctr for hereditary arrhythmic diseases, Cardiologic Dept & U1087 Nantes, France

*Equal first and last authors / †deceased

Correspondence:

Elijah R. Behr, MA, MBBS, MD, FRCP, FESC

Professor of Cardiovascular Medicine

Honorary Consultant Cardiologist and Electrophysiologist

Cardiology Clinical Academic Group

St. George's, University of London

St. George's University Hospitals NHS Foundation Trust

Tel: +442087252994

E-mail: ebehr@sgul.ac.uk

Journal Subject Terms: Sudden Cardiac Death; Pathophysiology; Genetics; Genetic, Association Studies

This article is published in its accepted form; it has not been copyedited and has not appeared in an issue of the journal. Preparation for inclusion in an issue of *Circulation: Genomic and Precision Medicine* involves copyediting, typesetting, proofreading, and author review, which may lead to differences between this accepted version of the manuscript and the final, published version.

Abstract:

Background - Brugada syndrome (BrS) is characterized by the type 1 Brugada ECG pattern. Pathogenic rare variants in *SCN5A* (mutations) are identified in 20% of BrS families in whom incomplete penetrance and genotype-negative phenotype-positive individuals are observed. E1784K-*SCN5A* is the most common *SCN5A* mutation identified. We determined the association of a BrS genetic risk score (BrS-GRS) and *SCN5A* mutation type on BrS phenotype in BrS families with *SCN5A* mutations.

Methods - Subjects with a spontaneous type 1 pattern or positive/negative drug challenge from cohorts harboring *SCN5A* mutations were recruited from 16 centers (n=312). Single nucleotide polymorphisms (SNP) previously associated with BrS at genome-wide significance were studied in both cohorts: rs11708996, rs10428132 and rs9388451. An additive linear genetic model for the BrS-GRS was assumed (6 SNP risk alleles).

Results - In the total population (n=312), BrS-GRS ≥4 risk alleles yielded an odds ratio (OR) of 4.15 for BrS phenotype (95%CI:1.45-11.85, p=0.0078). Amongst *SCN5A*-positive individuals (n=258), BrS-GRS ≥4 risk alleles yielded an odds ratio (OR) of 2.35 (95%CI:0.89-6.22, p=0.0846). In *SCN5A*-negative relatives (n=54), BrS-GRS ≥4 alleles yielded and OR of 22.29 (95%CI:1.84-269.30, p=0.0146). Among E1784K-*SCN5A* positive family members (n=79), hosting ≥4 risk alleles gave an OR=5.12 (95%CI:1.93-13.62, p=0.0011).

Conclusions - Common genetic variation is associated with variable expressivity of BrS phenotype in *SCN5A* families, explaining in part incomplete penetrance and genotype-negative phenotype-positive individuals. *SCN5A* mutation genotype and a BrS-GRS associate with BrS phenotype but the strength of association varies according to presence of a *SCN5A* mutation and severity of loss of function.

Key words: Brugada syndrome; genetics, human; risk score; SCN5A; single nucleotide polymorphism genetics

This article is published in its accepted form; it has not been copyedited and has not appeared in an issue of the journal. Preparation for inclusion in an issue of *Circulation: Genomic and Precision Medicine* involves copyediting, typesetting, proofreading, and author review, which may lead to differences between this accepted version of the manuscript and the final, published version.

DOI: 10.1161/CIRCGEN.120.002911

Nonstandard Abbreviations and Acronyms

ACMG American College of Medical Genetics

BrS Brugada syndrome

BrS-GRS Brugada syndrome genetic risk score

ECG Electrocardiogram

GEE Generalised estimating equation

GWAS Genome wide association study

ICC Inherited cardiac conditions

KASP Kompetitive Allele Specific PCR

OR Odds ratio

PCR Polymerase chain reaction

SD Standard deviation

SNP Single nucleotide polymorphism

American Heart Association.

Circulation: Genomic and Precision Medicine

Introduction

Brugada syndrome (BrS) is characterized by the type 1 Brugada ECG pattern, present either spontaneously or after provocation with a sodium channel blocking agent.[1] Pathogenic rare variants (mutations) in the *SCN5A* gene, encoding the Nav1.5 sodium channel, are identified in 20% of cases.[2,3] Incomplete penetrance and variable expression is common in BrS pedigrees with *SCN5A* mutations, suggesting a complex inheritance wherein other genetic variants may affect the phenotype.[2] Genotype-negative individuals from *SCN5A*-positive pedigrees have shown the type 1 Brugada ECG pattern.[2] Furthermore, common genetic variation has been associated with BrS in probands, independent of *SCN5A* status.[4]

The E1784K-*SCN5A* mutation (c.5350G>A; ClinVar ID: 9377) is the most common *SCN5A* mutation identified in BrS, identified in 3% of unrelated BrS cases[3,5] and is absent in the

This article is published in its accepted form; it has not been copyedited and has not appeared in an issue of the journal. Preparation for inclusion in an issue of *Circulation: Genomic and Precision Medicine* involves copyediting, typesetting, proofreading, and author review, which may lead to differences between this accepted version of the manuscript and the final, published version.

DOI: 10.1161/CIRCGEN.120.002911

gnomAD database. Furthermore, E1784K-*SCN5A* exhibits incomplete penetrance and can manifest as a mixed clinical phenotype of long QT syndrome and/or BrS, even amongst affected individuals from the same pedigree.[6,7] These properties make E1784K-*SCN5A* an optimal target for studying potential genetic modifiers.[8]

We hypothesized that common genetic variation previously associated with BrS[4], and a genetic risk score derived thereof (BrS-GRS), is associated with a type 1 Brugada ECG pattern in genotype-positive individuals from BrS families hosting *SCN5A* mutations as well as in genotype-negative relatives. We then explored the effects of *SCN5A* mutation type on the likelihood of a type 1 Brugada ECG pattern.

American Heart Association.

Methods

The data that support the findings of this study are available from the corresponding author upon reasonable request. IRB approval was obtained, according to the guidelines noted in Instructions to Authors. The full methods are available as supplemental data.

Circulation: Genomic and Precision Medicine

Results

Clinical characteristics

The total cohort comprised of 312 individuals from families harboring *SCN5A* mutations. The individuals that fulfilled inclusion criteria had the presence or absence of the BrS phenotype definitively established and had undergone complete SNP genotyping (Figure 1). These 312 individuals were recruited from 137 families. The median family size was 1 (Q1-Q3: 1-2); four families had between 10 and 20 individuals and a single family contributed 31 individuals.

This article is published in its accepted form; it has not been copyedited and has not appeared in an issue of the journal. Preparation for inclusion in an issue of *Circulation: Genomic and Precision Medicine* involves copyediting, typesetting, proofreading, and author review, which may lead to differences between this accepted version of the manuscript and the final, published version.

Figure 1 illustrates the breakdown of included cases according to *SCN5A* genotype and mutation type.

Clinical characteristics are described and compared in Table 1. Subjects hosting *SCN5A*-E1784K, when compared to individuals harboring loss-of-function mutations causing haploinsufficiency and other missense *SCN5A* mutations, were younger and more likely to be female. As would be expected when comparing individuals with an overlap syndrome to those with conduction disease, they exhibited longer QT intervals and shorter PR intervals and QRS durations on their presenting ECGs.

Seventy-nine individuals were E1784K-*SCN5A* positive. Fifty-seven (72%) with E1784K-*SCN5A* had BrS phenotype (10 spontaneous; 47 drug-induced). Amongst the 179 individuals harboring loss-of-function mutations causing haploinsufficiency and other missense *SCN5A* mutations, 164 (92%) had BrS phenotype (78 spontaneous; 61 drug-induced; 25 unspecified). Importantly, 6/54 (17%) *SCN5A* negative subjects displayed a drug-induced BrS phenotype. The associations of *SCN5A* mutation and/or BrS-GRS with the spontaneous BrS phenotype are similar to those described in both spontaneous and drug induced BrS combined, but were less accurate with higher p-values (data not shown).

American Heart Association.
Circulation: Genomic and Precision Medicine

SCN5A mutation associations (Figure 2)

Amongst *SCN5A* families the presence of an *SCN5A* mutation was associated with an odds ratio (OR) of 51.98 (95%CI:20.02-134.93, p<0.0001) for BrS phenotype. In all three *SCN5A* mutation type subgroups, i.e. E1784K-*SCN5A*, loss-of-function mutations causing haploinsufficiency and missense mutations other than E1784K-*SCN5A*, genotype positive patients were at an increased risk of BrS compared to genotype negative patients, but the odds ratios differed significantly ($p_{interaction}$=0.004) between the mutation types.

This article is published in its accepted form; it has not been copyedited and has not appeared in an issue of the journal. Preparation for inclusion in an issue of *Circulation: Genomic and Precision Medicine* involves copyediting, typesetting, proofreading, and author review, which may lead to differences between this accepted version of the manuscript and the final, published version.

Amongst *SCN5A* genotype positive individuals only, both loss-of-function mutations causing haploinsufficiency and other missense mutations had an increased risk of BrS compared to E1784K-*SCN5A* with OR = 6.11 (95%CI:1.78-20.97, p=0.0040) and OR = 3.44 (95%CI:1.35-8.75, p=0.0095), respectively.

Brugada Syndrome Genetic Risk Score

The BrS-GRS was calculated for each subject in the total cohort as described. A weighted BrS-GRS was also tested, but this did not outperform the non-weighted BrS-GRS (data not shown). Figure 3 shows the distribution of proportion of subjects according to numbers of risk alleles (range 0-6) for the total cohort and subsets of *SCN5A* mutations. In the total population, the odds ratio per allele was 1.46 (95%CI 1.11-1.94, p=0.0076) and individuals with a BrS-GRS ≥4 risk alleles had an OR=4.15 (95%CI 1.45-11.85, p=0.0078) for BrS phenotype compared to individuals with a GRS <4 risk alleles.

American Heart Association.
Circulation: Genomic and Precision Medicine

The BrS-GRS effects per allele and ≥4 risk alleles appeared smaller in *SCN5A* genotype positives, but this was not significant ($p_{interaction}$ = 0.090 and 0.076, respectively). Within *SCN5A* genotype positives only, the BrS-GRS effects per allele and ≥4 risk alleles were significantly different between mutation types ($p_{\text{interaction}}$ = 0.0096 and <0.0001, respectively).

SCN5A genotype-positive relatives (n=258) yielded an OR=1.25 (95%CI 0.92-1.71, p=0.1571) for BrS phenotype per risk allele. Individuals with a BrS-GRS ≥4 risk alleles had an OR=2.35 (95%CI:0.89-6.22, p=0.0846) for BrS phenotype compared to individuals with a GRS <4 risk alleles. *SCN5A* genotype-negative relatives (n=54) yielded an OR for BrS phenotype of 2.71 per risk allele (95%CI 0.98-7.43, p=0.0535). *SCN5A* genotype-negative individuals with a BrS-GRS ≥4 risk alleles had an OR=22.29 (95%CI 1.84-269.30, p=0.0146) for BrS phenotype compared to individuals with a BrS-GRS <4 risk alleles (Figures 4 and 5).

This article is published in its accepted form; it has not been copyedited and has not appeared in an issue of the journal. Preparation for inclusion in an issue of *Circulation: Genomic and Precision Medicine* involves copyediting, typesetting, proofreading, and author review, which may lead to differences between this accepted version of the manuscript and the final, published version.

SCN5A loss-of-function mutations causing haploinsufficiency

For subjects hosting loss-of-function *SCN5A* mutations causing haploinsufficiency the association between the BrS-GRS and BrS phenotype appeared the strongest (OR per risk allele of 5.18; 95%CI:2.07-12.93, p=0.0004). Since there were no BrS negative cases that had more than 2 risk alleles, the OR of subjects with ≥4 risk alleles was infinite compared to subjects with <4 risk alleles (Figures 3, 4 and 5).

SCN5A-E1784K

When examining E1784K-*SCN5A* positive family members alone, a weaker BrS-GRS performance was found: OR=1.49 (95%CI 1.09-2.04, p=0.0135) per risk allele. Individuals with a BrS-GRS ≥4 risk alleles had an OR=5.12 (95%CI:1.93-13.62, p=0.0011) for BrS phenotype compared to individuals with a GRS <4 risk alleles (Figures 4 and 5).

Other missense SCN5A mutations

For individuals hosting other *SCN5A* mutations there was no statistically significant association between the BrS-GRS and BrS phenotype (OR per risk allele=0.88, 95%CI 0.58-1.32, p=0.5271). Subjects with ≥4 risk alleles had an OR=1.03 (95%CI:0.20-5.35, p=0.9705) for BrS phenotype compared to those with less than 4 risk alleles (Figures 4 and 5).

Circulation: Genomic and Precision Medicine

Discussion

Historically, BrS was considered an autosomal dominant monogenic disorder. In 2013, a common variant GWAS comparing index cases of BrS to healthy controls indicated association with common genetic variation, regardless of presence of an *SCN5A* mutation.[4] While that work identified susceptibility loci, up to now, the variable expression of the BrS phenotype in members of families with *SCN5A* mutations has remained unexplained. Here, for the first time

This article is published in its accepted form; it has not been copyedited and has not appeared in an issue of the journal. Preparation for inclusion in an issue of *Circulation: Genomic and Precision Medicine* involves copyediting, typesetting, proofreading, and author review, which may lead to differences between this accepted version of the manuscript and the final, published version.

we report that common genetic variation, in the form of a BrS-GRS, correlates with the BrS phenotype in individuals from families with *SCN5A* loss-of-function mutations causing haploinsufficiency and the recurrent mutation E1784K-*SCN5A*. Furthermore, our study extends beyond the findings of the original GWAS by emphasizing the role of common variation in expression of the BrS phenotype independent of the presence of an *SCN5A* mutation. The BrS-GRS explained in part the variable expression of BrS phenotype in both *SCN5A*-positive and *SCN5A*-negative relatives. There was significant heterogeneity of the strength of association of different types of *SCN5A* mutation (loss-of-function causing haploinsufficiency, E1784K and other missense) and their associated BrS-GRS with BrS phenotype indicating a variable biological effect of common and rare variants on disease susceptibility. These findings support a complex polygenic architecture for BrS and are an important proof of principle in cardiac genetics.

Circulation: Genomic and Precision Medicine

A BrS-GRS and variability in BrS phenotype within affected families

We sought to investigate whether a BrS-GRS is associated with BrS phenotype. The score demonstrated association with BrS phenotype in pedigrees carrying pathogenic or likely pathogenic *SCN5A* variants, reflecting the cumulative effect of the three SNPs (six risk alleles) on BrS phenotype. The BrS-GRS was then tested separately in the subset of families harboring loss-of-function *SCN5A* mutations causing haploinsufficiency, detecting a strong effect size and a near infinite OR when harboring four or more risk alleles. This may reflect the small numbers of Brugada negative cases with loss-of-function mutations causing haploinsufficiency and that chromosome 3 risk alleles in *trans* with the mutant allele are more likely to have a more potent effect by further altering the expression of already haplo-insufficient wild-type *SCN5A*.

This article is published in its accepted form; it has not been copyedited and has not appeared in an issue of the journal. Preparation for inclusion in an issue of *Circulation: Genomic and Precision Medicine* involves copyediting, typesetting, proofreading, and author review, which may lead to differences between this accepted version of the manuscript and the final, published version.

Families with missense *SCN5A* mutations other than E1784K-*SCN5A* showed no significant associations with the BrS-GRS whilst the E1784K-*SCN5A* subset exhibited a significant association, albeit weaker than for loss-of-function mutations causing haploinsufficiency. The reasons for this difference are likely to be complex. Firstly, E1784K-*SCN5A* is considered a relatively mild missense mutation in its biophysical and clinical consequences and showed lower penetrance in our study compared to other missense mutations (72% vs 90% respectively, Table 1).[9] The association of the BrS-GRS may therefore reflect a greater impact of common variation in this setting. Secondly, the diversity of the other included missense *SCN5A* mutations may have led to a weaker power for evaluating the BrS-GRS compared to E1784K-SCN5A families. Each mutation is expected to have different severity of biophysical defects with the potential for variable effects of SNPs on the lesion. Furthermore, due to the small size and heterogeneity of the total cohort, there was insufficient power to analyze chromosomal phasing between *SCN5A* mutations and the SNPs of interest. The other missense *SCN5A* mutation group was therefore a less homogeneous group to test for associations than a large group of families with a single mutation such as E1784K-*SCN5A*. More homogeneous samples, particularly founder populations, may be more appropriate for future studies of how common variants modify phenotype. Interestingly in *SCN5A* genotype-negative relatives, the association of BrS-GRS ≥4 risk alleles with BrS phenotype was even more apparent. In fact, the OR was greater than that of E1784K-*SCN5A* in isolation. This supports a greater strength of association of common variation with the likelihood of BrS phenotype in the absence of a *SCN5A* mutation.

These results therefore reveal the potential for clinical utility of incorporating common genetic variation in the form of a genetic risk score in genetic diagnostics for rare disease. It is

American Heart Association.

Circulation: Genomic and Precision Medicine

This article is published in its accepted form; it has not been copyedited and has not appeared in an issue of the journal. Preparation for inclusion in an issue of *Circulation: Genomic and Precision Medicine* involves copyediting, typesetting, proofreading, and author review, which may lead to differences between this accepted version of the manuscript and the final, published version.

expected, however, that additional SNPs underlie the complex genetic nature of BrS and a larger GWAS is needed to identify other common variants that could be incorporated to improve the power of an optimized BrS-GRS for diagnostic purposes. This will also require further investigation of greater numbers of relatives with integration of haplotype structure and detailed knowledge of *SCN5A* variants' biophysical properties.

Association of rare *SCN5A* variation with BrS phenotype and common variants

While common variation in the form of a BrS-GRS has clear independent association with BrS phenotype, the strongest contribution comes from the presence of an *SCN5A* mutation. However, not all *SCN5A* BrS susceptibility mutations have comparable functional effects. The OR for the BrS phenotype associated with E1784K-*SCN5A* is significantly lower than for other missense *SCN5A* mutations but is greatest in loss-of-function mutations causing haploinsufficiency. Furthermore, the OR of the BrS-GRS for BrS phenotype varied according to *SCN5A* mutation and was strongest in genotype negative relatives. This suggests that there may be an interaction and synergy of common and rare variation affecting sodium channel function whereby a certain level of impairment is necessary to achieve a threshold where BrS phenotype can manifest. This further supports a polygenic genetic architecture underlying the condition.[10]

American Heart Association.
Circulation: Genomic and Precision Medicine

Genotype-phenotype mismatch in BrS and its implications

The proposed polygenic model of heritability in BrS may explain the paradox of clinically affected mutation-negative individuals in *SCN5A* families, first demonstrated by Probst et al.[2] Indeed, 12% of *SCN5A*-negative relatives showed a drug-induced BrS phenotype. Importantly, cascade genetic screening in *SCN5A* pedigrees can result in *SCN5A* genotype-negative relatives being discharged from further follow-up. A small proportion of these individuals may still be at risk of developing a BrS phenotype. Conversely, these findings also raise further questions about

This article is published in its accepted form; it has not been copyedited and has not appeared in an issue of the journal. Preparation for inclusion in an issue of *Circulation: Genomic and Precision Medicine* involves copyediting, typesetting, proofreading, and author review, which may lead to differences between this accepted version of the manuscript and the final, published version.

the specificity of drug provocation tests for BrS in the absence of a gold standard test for the condition. The prevalence of the type 1 Brugada ECG pattern after drug provocation testing has already been shown to be much higher than expected (4%) in healthy controls.[11] Indeed recent data have associated a similar BrS polygenic risk score with the ajmaline induced type 1 pattern.[12] The Shanghai consensus document downgraded the diagnostic certainty offered by such a result when found in isolation.[13] The likelihood of a drug-induced type 1 Brugada ECG pattern indicating a diagnosis of BrS is considered greater, however, if an individual had a family history of premature autopsy negative SCD and/or BrS. The significance of a drug-induced type 1 Brugada ECG pattern in *SCN5A* genotype-negative relatives is therefore uncertain in *SCN5A* BrS families. Other, as yet unknown, polygenic and acquired contributions to the risk of developing BrS phenotype may be present in these *SCN5A* genotype-negative relatives.

American Heart Association.

BrS phenotype-positive *SCN5A* genotype-negative individuals may be identified due to clinical evaluation taking place either prior to genetic studies being available, or prior to determination of the pathogenicity of a detected rare *SCN5A* variant. There is insufficient follow-up data available, however, in the literature to determine if these individuals subsequently develop arrhythmic events. In the meantime, these patients may be offered monitoring for evidence of evolving risk and lifestyle advice such as avoidance of prescription sodium channel blocking drugs, cocaine and alcohol intoxication, and treatment of fever.[14] Asymptomatic *SCN5A*-negative relatives of autopsy-negative SCD victims, who go on to have a positive ajmaline test, have been managed with this strategy. During follow-up, a spontaneous type 1 Brugada ECG pattern and/or clinically significant arrhythmic events developed in 17% of these individuals.[15] This may be a worthwhile approach in BrS *SCN5A* family members, regardless of genotype status, although further prospective research will be required.

Circulation: Genomic and Precision Medicine

This article is published in its accepted form; it has not been copyedited and has not appeared in an issue of the journal. Preparation for inclusion in an issue of *Circulation: Genomic and Precision Medicine* involves copyediting, typesetting, proofreading, and author review, which may lead to differences between this accepted version of the manuscript and the final, published version.

DOI: 10.1161/CIRCGEN.120.002911

Future perspective: an optimized BrS-GRS

There is already strong association of a BrS-GRS ≥4 risk alleles utilizing only three SNPs with BrS phenotype. We propose that an optimized BrS-GRS employing additional SNPs emerging from a larger GWAS could act as a complementary approach to quantifying the probability of developing BrS phenotype. Furthermore, incorporating phasing of SNPs could further refine the predictive accuracy of a BrS-GRS, especially in *SCN5A* families where SNPs in *trans* to the *SCN5A* mutant allele would be expected to have more pronounced effects than SNPs in *cis*. An optimized and validated GRS may therefore aid decision-making over follow-up in *SCN5A* families and determining whether preventative and monitoring strategies for BrS should be instituted.[1,13] A GRS-based approach may even replace the unnecessary use of drug challenge and form part of clinical genetic testing in BrS.

American Heart Association.

Limitations

Circulation: Genomic and Precision Medicine

BrS phenotype was defined in accordance with the 2013 HRS/EHRA/APHRS guidelines. Other guidelines have been proposed due to concerns over the specificity of the sodium channel blocker-induced BrS phenotype.[1,13,15] These guidelines maintain the same definition of the type 1 Brugada ECG pattern and give extra weight to a family history of BrS. We therefore used the same ECG definition for BrS phenotype in this study. We also treated spontaneous and drug-induced BrS phenotype as one group for analysis purposes. This was due to low numbers, the similarity of findings in spontaneous BrS (data not shown) as well as the consistency of the association demonstrated by the BrS GWAS regardless of whether the phenotype was drug-induced or spontaneous.[4]

A smaller proportion of the *SCN5A* genotype-negative cases underwent sodium channel blocker challenge, probably reflecting variation in local clinical practice. Furthermore, only a

This article is published in its accepted form; it has not been copyedited and has not appeared in an issue of the journal. Preparation for inclusion in an issue of *Circulation: Genomic and Precision Medicine* involves copyediting, typesetting, proofreading, and author review, which may lead to differences between this accepted version of the manuscript and the final, published version.

relatively small proportion of *SCN5A* genotype-positive relatives were found to be BrS phenotype-negative after drug challenge. Both factors likely weakened the power to detect associations.

Due to the heterogeneity of the total cohort, there was insufficient power to analyze chromosomal phasing between the *SCN5A* mutations and the SNPs of interest at this chromosomal locus - rs11708996 (*SCN5A*) and rs10428132 (*SCN10A*) - and therefore SNP interactions. These potential interactions may explain why the weighted model for the BrS-GRS did not show additional significance over the additive model. Furthermore, families of Japanese and other non-Caucasian ancestry were included but due to small numbers could not be analysed separately. This was offset, however, by the three SNPs used to create the BrS-GRS having been replicated in Japanese BrS cases[16].

American Heart Association.

Conclusions

Circulation: Genomic and Precision Medicine

Common genetic variation explains in part, the variable expression of BrS phenotype in families with sodium channel disease. Association of common variants was cumulative leading to a BrS-GRS associated with BrS phenotype in both genotype positive and negative subjects i.e. independent of the presence of an *SCN5A* mutation. *SCN5A* mutations and the BrS-GRS also show differing effect sizes on BrS phenotype according to variant type, further confirming a complex polygenic architecture underlying BrS. These findings have important implications in BrS *SCN5A* families where a *SCN5A*-negative relative may still develop a BrS phenotype. Further work is required to elucidate other genetic factors to develop an optimized BrS-GRS that may become a surrogate marker for BrS phenotype in *SCN5A* families, form part of clinical genetic testing, obviate drug provocation testing and guide follow-up.

Acknowledgments: In memory of our dear colleague and friend Velislav Batchvarov.

This article is published in its accepted form; it has not been copyedited and has not appeared in an issue of the journal. Preparation for inclusion in an issue of *Circulation: Genomic and Precision Medicine* involves copyediting, typesetting, proofreading, and author review, which may lead to differences between this accepted version of the manuscript and the final, published version.

Sources of Funding: This work was supported by James Lancaster Memorial Fund sponsored by McColl's RG Ltd. YDW and ERB acknowledge funding and ongoing support from the James Lancaster Memorial Fund sponsored by McColl's RG Ltd. YDW had received support through an Academic Clinical Fellowship from the National Institute of Health Research. ERB is supported by the Higher Education Funding Council for England and the British Heart Foundation (BHF). ERB, MM, HR, MP and SS acknowledge support from Cardiac Risk in the Young. ERB and HR acknowledge support from BHF Project Grant PG/15/107/31908 and BHF Clinical Research Training Fellowship FS/11/71/28918 respectively. MM acknowledges support from the Medical Research Council. CRB and AAW acknowledge the support from the Dutch Heart Foundation (CVON PREDICT2 project to CRB, HLT and AAW) and the Netherlands Organization for Scientific Research (VICI fellowship, 016.150.610, to CRB). RT received support from the Philippa and Marvin Carsley Cardiology Chair, and is currently a clinical research scholar of the Fonds de Recherche du Québec – Santé. DJT, MJB and MJA are supported by the Mayo Clinic Windland Smith Rice Comprehensive Sudden Cardiac Death Program.

Circulation: Genomic and Precision Medicine

Disclosures: ERB received prior research funds from Biotronik and consulting for Medtronic. MJA reports involvement. AliveCor, Audentes Therapeutics, Blue Ox Health, Boston Scientific, Gilead Sciences, Invitae, Medtronic, MyoKardia, StemoniX, and St. Jude Medical. All others have none.

References:

1. Priori SG, Wilde AA, Horie M, Cho Y, Behr ER, Berul C, Blom N, Brugada J, Chiang C-E, Huikuri H, et al. HRS/EHRA/APHRS expert consensus statement on the diagnosis and management of patients with inherited primary arrhythmia syndromes: document endorsed by HRS, EHRA, and APHRS in May 2013 and by ACCF, AHA, PACES, and AEPC in June 2013. *Heart Rhythm*. 2013;10:1932–1963.

2. Probst V, Wilde AAM, Barc J, Sacher F, Babuty D, Mabo P, Mansourati J, Le Scouarnec S, Kyndt F, Le Caignec C, et al. SCN5A mutations and the role of genetic background in the pathophysiology of Brugada syndrome. *Circ Cardiovasc Genet*. 2009;2:552–7.

3. Kapplinger JD, Tester DJ, Alders M, Benito B, Berthet M, Brugada J, Brugada P, Fressart V, Guerchicoff A, Harris-Kerr C, et al. An international compendium of mutations in the SCN5A-encoded cardiac sodium channel in patients referred for Brugada syndrome genetic testing. *Heart Rhythm*. 2010;7:33–46.

This article is published in its accepted form; it has not been copyedited and has not appeared in an issue of the journal. Preparation for inclusion in an issue of *Circulation: Genomic and Precision Medicine* involves copyediting, typesetting, proofreading, and author review, which may lead to differences between this accepted version of the manuscript and the final, published version.

4. Bezzina CR, Barc J, Mizusawa Y, Remme CA, Gourraud J-B, Simonet F, Verkerk AO, Schwartz PJ, Crotti L, Dagradi F, et al. Common variants at SCN5A-SCN10A and HEY2 are associated with Brugada syndrome, a rare disease with high risk of sudden cardiac death. *Nat Genet*. 2013;45:1044–1049.

5. Tester DJ, Will ML, Haglund CM, Ackerman MJ. Compendium of cardiac channel mutations in 541 consecutive unrelated patients referred for long QT syndrome genetic testing. *Heart Rhythm*. 2005;2:507–517.

6. Makita N, Behr E, Shimizu W, Horie M, Sunami A, Crotti L, Schulze-Bahr E, Fukuhara S, Mochizuki N, Makiyama T, et al. The E1784K mutation in SCN5A is associated with mixed clinical phenotype of type 3 long QT syndrome. *J Clin Invest*. 2008;118:2219–29.

7. Veltmann C, Barajas-Martinez H, Wolpert C, Borggrefe M, Schimpf R, Pfeiffer R, Cáceres G, Burashnikov E, Antzelevitch C, Hu D. Further Insights in the Most Common SCN5A Mutation Causing Overlapping Phenotype of Long QT Syndrome, Brugada Syndrome, and Conduction Defect. *J Am Heart Assoc*. 2016;5:pii: e003379.

8. Schwartz PJ, Crotti L, George AL. Modifier genes for sudden cardiac death. *Eur Heart J*. 2018;39:3925–3931.

9. Baruteau A-E, Kyndt F, Behr ER, Vink AS, Lachaud M, Joong A, Schott J-J, Horie M, Denjoy I, Crotti L, et al. SCN5A mutations in 442 neonates and children: genotype-phenotype correlation and identification of higher-risk subgroups. *Eur Heart J*. 2018;39:2879–2887.

10. Schwartz PJ, Gentilini D. Can genetics predict risk for sudden cardiac death? The relentless search for the Holy Grail. *Eur Heart J*. 2018;39:3970–3972.

11. Hasdemir C, Payzin S, Kocabas U, Sahin H, Yildirim N, Alp A, Aydin M, Pfeiffer R, Burashnikov E, Wu Y, et al. High prevalence of concealed Brugada syndrome in patients with atrioventricular nodal reentrant tachycardia. *Heart Rhythm*. 2015;12:1584–94.

12. Tadros R, Tan HL, ESCAPE-NET Investigators, El Mathari S, Kors JA, Postema PG, Lahrouchi N, Beekman L, Radivojkov-Blagojevic M, Amin AS, et al. Predicting cardiac electrical response to sodium-channel blockade and Brugada syndrome using polygenic risk scores. *Eur Heart J*. 2019;40:3097–3107.

13. Antzelevitch C, Yan G-X, Ackerman MJ, Borggrefe M, Corrado D, Guo J, Gussak I, Hasdemir C, Horie M, Huikuri H, et al. J-Wave syndromes expert consensus conference report: Emerging concepts and gaps in knowledge. *Heart Rhythm*. 2016;13:e295-324.

14. Priori SG, Pandit S V, Rivolta I, Berenfeld O, Ronchetti E, Dhamoon A, Napolitano C, Anumonwo J, di Barletta MR, Gudapakkam S, et al. A novel form of short QT syndrome (SQT3) is caused by a mutation in the KCNJ2 gene. *Circ Res*. 2005;96:800–807.

15. Papadakis M, Papatheodorou E, Mellor G, Raju H, Bastiaenen R, Wijeyeratne Y, Wasim S, Ensam B, Finocchiaro G, Gray B, et al. The Diagnostic Yield of Brugada Syndrome After Sudden Death With Normal Autopsy. *J Am Coll Cardiol*. 2018;71:1204–1214.

16. Bethesda (MD): National Center for Biotechnology Information NL of M. Database of Single Nucleotide Polymorphisms (dbSNP). dbSNP Build ID 151. [cited 2020 Sep 17]. Available from: https://www.ncbi.nlm.nih.gov/snp

This article is published in its accepted form; it has not been copyedited and has not appeared in an issue of the journal. Preparation for inclusion in an issue of *Circulation: Genomic and Precision Medicine* involves copyediting, typesetting, proofreading, and author review, which may lead to differences between this accepted version of the manuscript and the final, published version.

Table 1. Clinical characteristics of (a) the total cohort broken down by genotype status; (b) comparing families harboring loss-of-function causing haploinsufficiency, other missense mutations and E1784K-*SCN5A*.

a)

	**Total population, n=312**		***SCN5A* genotype positive, n=258**		***SCN5A* genotype negative, n=54**		**p-value**
	**n**	**%**	**n**	**%**	**N**	**%**	
**Male**	169	54	143	55	26	48	0.3603
**Caucasian**	270	87	237	92	33	61	**0.0015**
**BrS**	227	73	221	86	6	11	**2.0E-16**
**Spontaneous BrS ECG pattern ***	88/201	44	88/196	45	0/5	0	**2.0E-16**
**Mutation type:**							
E1784K	103	33	79	31	24	44	0.2700†
LOF	79	25	62	24	17	31	
Missense	130	42	117	45	13	24	
**Quantitative variables**							
	**mean**	**SD**	**mean**	**SD**	**mean**	**SD**	
**Age at ECG**	38	17	39	17	35	16	0.3900
**PR interval**	186	38	192	38	159	42	**1.8E-12**
**QRS duration**	101	21	104	20	84	20	**1.4E-09**
**QTc interval**	425	43	429	45	410	31	**0.0064**

b)

	**Mutation type**						
	**Loss-of-function causing haploinsufficiency n=62**		**Missense (excluding *SCN5A*-E1784K) n=117**		***SCN5A*-E1784K n=79**		**p-value**
	**n**	**%**	**n**	**%**	**n**	**%**	
**Male**	44	71	68	58	31	39	**0.0001**
**Caucasian**	62	100	117	100	58	73	**2.0E-16**
**BrS**	59	95	105	90	57	72	**0.0007**
**Spontaneous BrS ECG pattern ***	31/50	62	47/89	53	10/57	18	**0.0011**
**Quantitative variables**							
	**mean**	**SD**	**mean**	**SD**	**mean**	**SD**	
**Age at ECG**	36	16	44	15	33	17	**0.0002**
**PR interval**	206	42	202	35	167	27	**3.7E-13**
**QRS duration**	112	20	104	21	98	14	**0.0003**
**QTc interval**	402	31	408	33	479	22	**2.0E-16**

BrS = Brugada syndrome; QTc interval = QT interval corrected by Bazzett's formula. * In 26 cases (25 genotype positive) specific data on the spontaneity of the type 1 pattern were missing. † overall p-value (chi-square test) testing the distribution of the three mutation types among *SCN5A* genotype positive vs. *SCN5A* genotype negative individuals.

Circulation: Genomic and Precision Medicine

This article is published in its accepted form; it has not been copyedited and has not appeared in an issue of the journal. Preparation for inclusion in an issue of *Circulation: Genomic and Precision Medicine* involves copyediting, typesetting, proofreading, and author review, which may lead to differences between this accepted version of the manuscript and the final, published version.

Figure Legends:

Figure 1. Flow diagram summarizing inclusion and numbers of individuals separated by genotype and BrS phenotype in each cohort.

Figure 2. Risk of Brugada Syndrome in patients carrying an *SCN5A* mutation, Loss-of-function mutations causing haploinsufficiency, missense mutations other than *SCN5A*, and E1784K-*SCN5A*. The odds ratio (OR) and 95% confidence interval for each mutation type are shown (adjusted for sex and age). The p-values denote the levels of significance of the odds ratios for Brugada Syndrome comparing each cohort to negative genotype using GEE.

American Heart Association.
Circulation: Genomic and Precision Medicine

Figure 3. Cumulative number of risk alleles at the three loci and the associated likelihood of BrS phenotype showing performance of the BrS-GRS for prediction of BrS phenotype in mutation positive individuals in the (a) total cohort; (b) individuals from families harboring E1784K-*SCN5A*); (c) individuals from families harboring loss-of-function *SCN5A* mutations causing haploinsufficiency; (d) other missense *SCN5A* mutations. Distribution of numbers of risk alleles hosted by individuals with BrS phenotype (black bars) in each cohort are shown vs family members ascertained to be BrS phenotype-negative (white bars). Each bar represents the proportion of individuals carrying the corresponding number of risk alleles as a percentage of the total number of individuals with the corresponding phenotype, i.e. denominator for the white bars being the total number of individuals with no BrS within the cohort, and the denominator for the black bars being the total number of individuals with BrS within the cohort.

This article is published in its accepted form; it has not been copyedited and has not appeared in an issue of the journal. Preparation for inclusion in an issue of *Circulation: Genomic and Precision Medicine* involves copyediting, typesetting, proofreading, and author review, which may lead to differences between this accepted version of the manuscript and the final, published version.

DOI: 10.1161/CIRCGEN.120.002911

Figure 4. Risk per additional risk allele in a linear model in the total cohort; genotype negative individuals; genotype positive individuals from families harboring loss-of-function mutations causing haploinsufficiency; genotype positive individuals from families harboring E1784K-*SCN5A*; genotype positive individuals from families harboring other missense *SCN5A* mutations. The odds ratio (OR) per additional risk allele and 95% confidence interval are shown (adjusted for sex and age). The p-values denote the levels of significance of the odds ratios per additional risk allele for Brugada Syndrome in each cohort using GEE. The OR and 95%CI for Genotype positive: Loss-of-function causing haploinsufficiency cohort are not shown as these are off the scale of this figure.

American Heart Association.

Figure 5. Risk of Brugada Syndrome in patients carrying ≥4 risk alleles in the total cohort; genotype negative individuals; genotype positive individuals from families harboring loss-of-function mutations causing haploinsufficiency; genotype positive individuals from families harboring E1784K-SCN5A; genotype positive individuals from families harboring other missense SCN5A mutations. The odds ratio (OR) and 95% confidence interval for a cut off of ≥4 risk alleles are shown (adjusted for age and sex). The p-values denote the level of significance of the odds ratios for this cut-off for Brugada Syndrome for each cohort using GEE.

Circulation: Genomic and Precision Medicine

This article is published in its accepted form; it has not been copyedited and has not appeared in an issue of the journal. Preparation for inclusion in an issue of *Circulation: Genomic and Precision Medicine* involves copyediting, typesetting, proofreading, and author review, which may lead to differences between this accepted version of the manuscript and the final, published version.

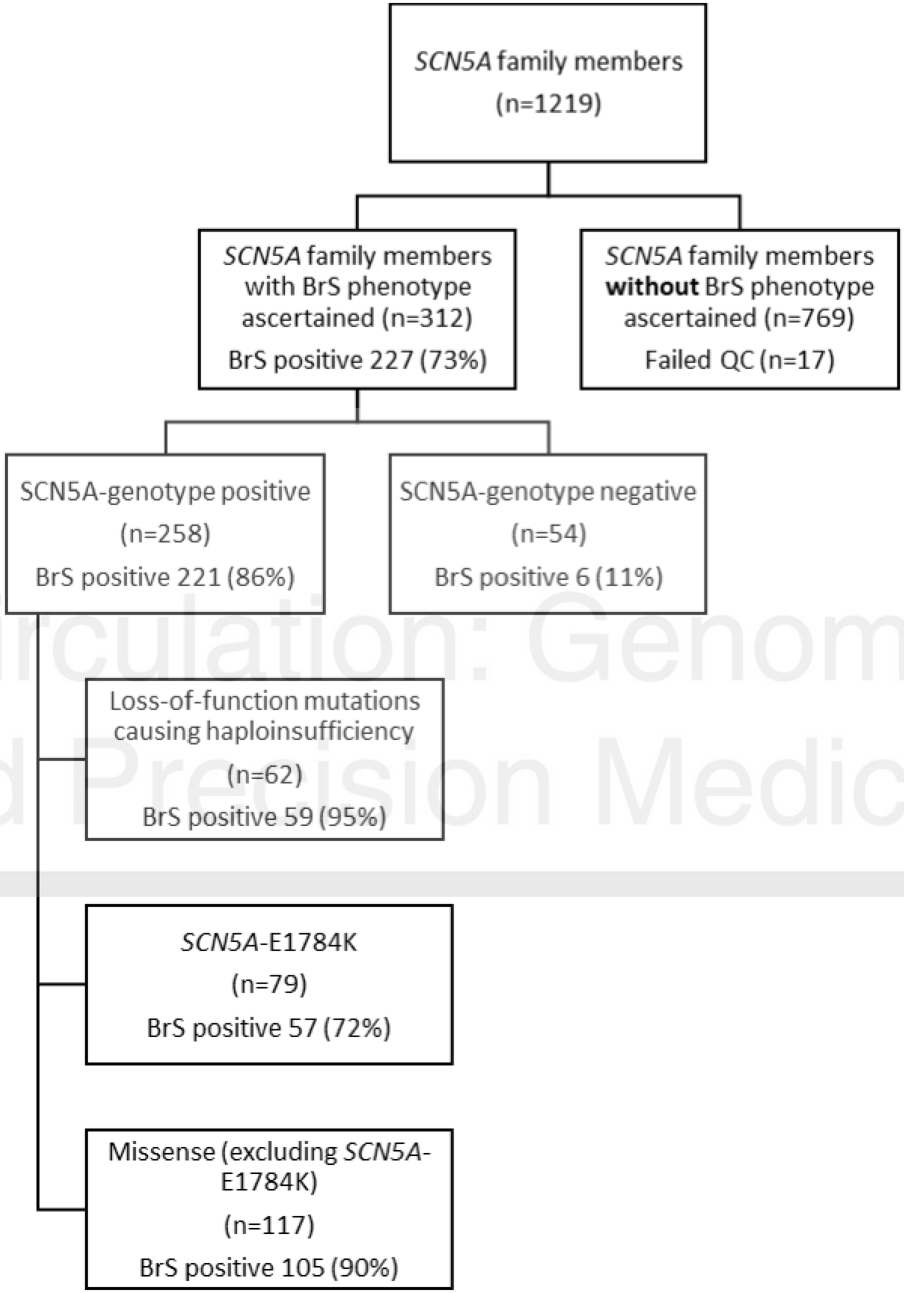

American Heart Association.
Circulation: Genomic and Precision Medicine

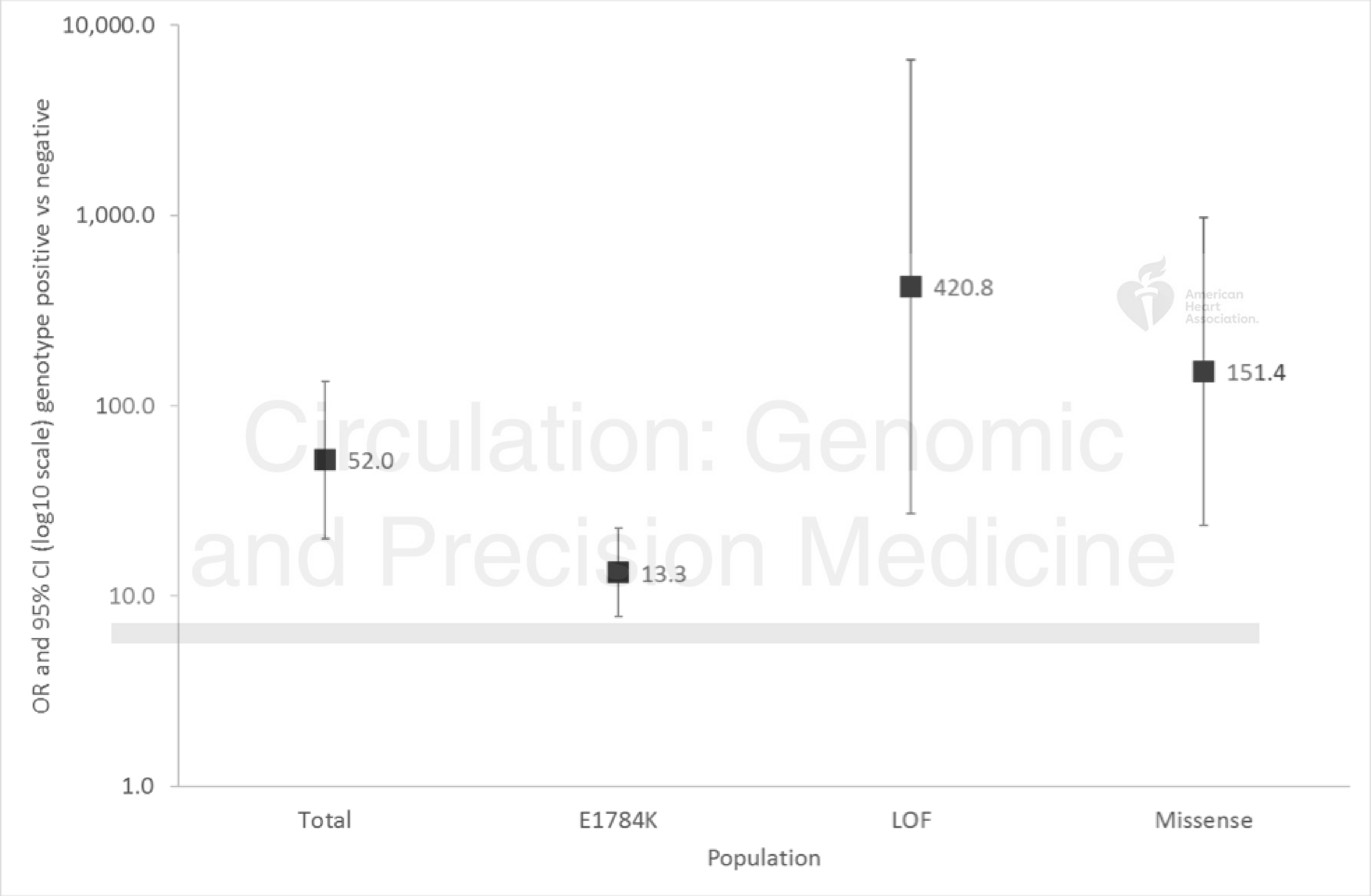

Circulation: Genomic and Precision Medicine

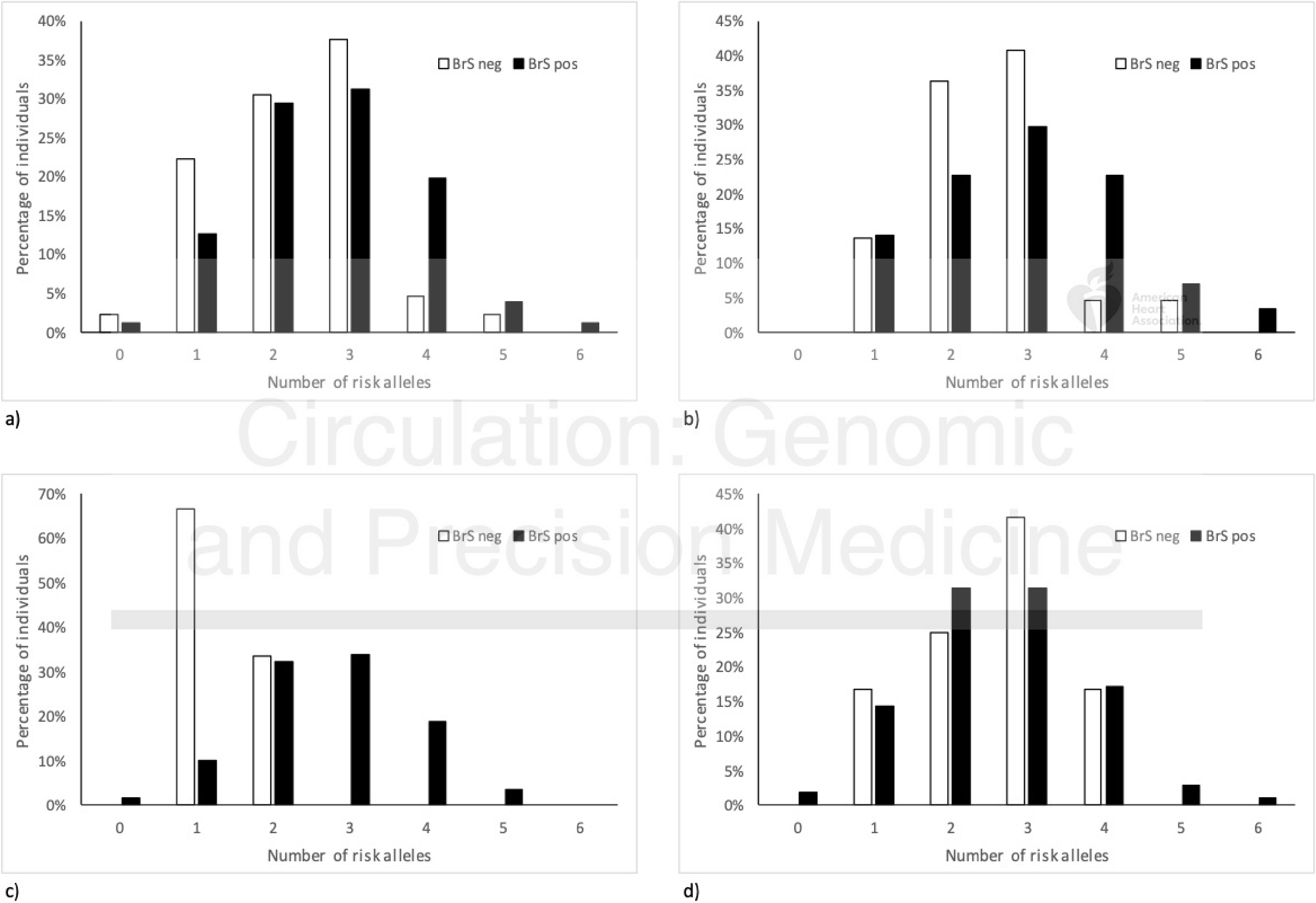

Circulation: Genomic and Precision Medicine

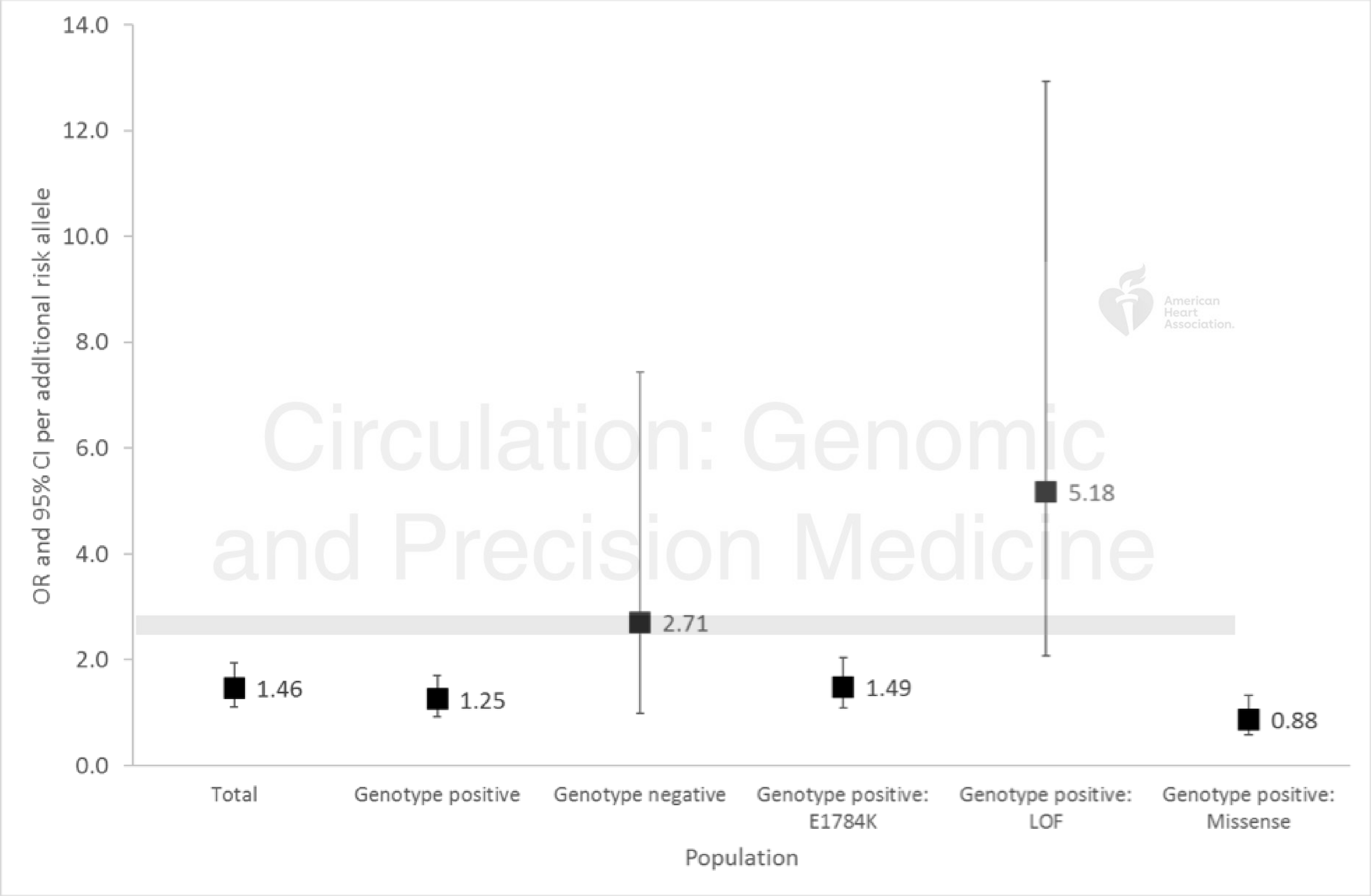

Circulation: Genomic and Precision Medicine

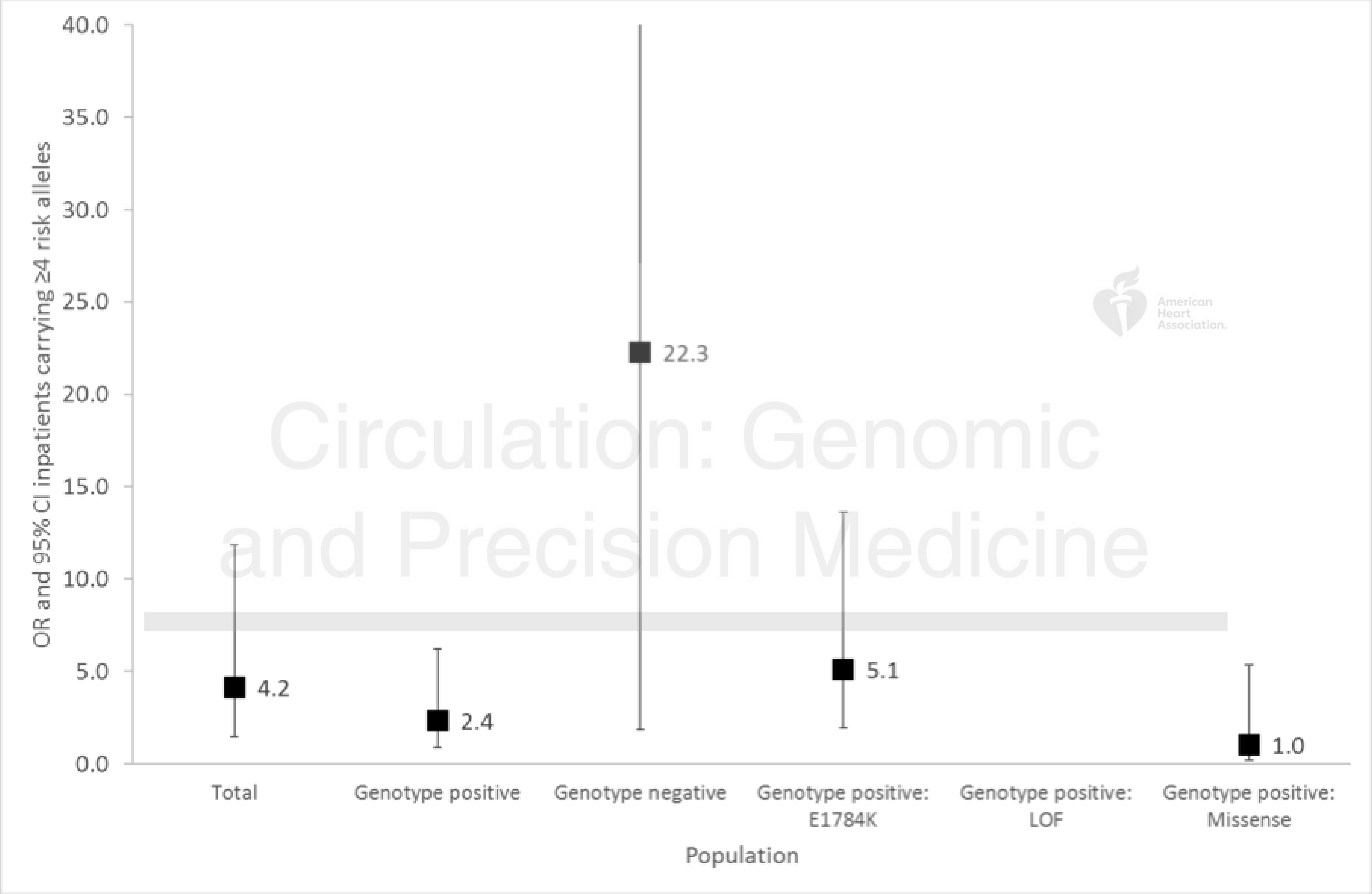

Circulation: Genomic and Precision Medicine
American Heart Association.
